# Comparative Life Cycle Assessment of Prussian White and NVP/C‐Based Sodium‐Ion Batteries Based on Primary Laboratory Data

**DOI:** 10.1002/cssc.202500268

**Published:** 2025-08-19

**Authors:** Friedrich B. Jasper, Manuel J. Baumann, Hüseyin Ersoy, Anna Smith, Sebastian Büchele, Nicole Bohn, Joachim R. Binder, Dirk Holger Neuhaus, Marcel Weil

**Affiliations:** ^1^ Institute for Technology Assessment and Systems Analysis (ITAS) KIT 76021 Karlsruhe Germany; ^2^ Department of Sustainable Systems Engineering (INATECH) University Freiburg 79110 Germany; ^3^ Institute for Applied Materials (IAM) KIT 76344 Karlsruhe Germany; ^4^ Fraunhofer Institute for Solar Energy Systems (ISE) Fraunhofer‐Gesellschaft Heidenhofstrasse 2 79110 Freiburg Germany; ^5^ Helmholtz‐Institute for Electrochemical Energy Storage (HIU) KIT 89081 Ulm Germany

**Keywords:** life cycle assessment, primary data, prussian white, sodium vanadium phosphate, sodium‐ion battery

## Abstract

Sodium‐ion batteries (SIBs) are considered the most promising candidate for electrochemical storage after lithium‐ion batteries (LIBs) to meet the globally growing energy storage demand. Assessments to identify environmental hotspots and address them in further development at regular intervals are inevitable to ensure low environmental impact of SIBs in the future. However, the number of studies assessing the environmental impacts of SIBs is limited, and existing studies are mostly based on theoretical models and few limited sources. This study is one of the few to provide life cycle inventory data from actual production processes of SIBs, allowing for hotspot analysis of environmental impacts. A comparative life cycle assessment of two emerging SIB technologies is conducted based on primary laboratory data, focusing on the synthesis of the two cathode active materials (CAMs), sodium vanadium phosphate (NVP/C), and a Prussian white (PW). The results show that the impacts of NVP/C‐based pouch cells are significantly higher than the PW‐based cells in all impact categories due to lower gravimetric energy densities and higher CAM synthesis‐related impacts. To ensure the correct order of magnitude a benchmark NMC‐based LIB is assessed at the same laboratory, showing lower impacts than both SIBs, due to higher gravimetric energy densities.

## Graphical abstract

This study provides insides on the environmental impacts of the lab‐scale production of two different sodium‐ion batteries (SIBs). It highlights the hotspots and is one of the few to provide primary life cycle inventory data on the production processes for SIBs.

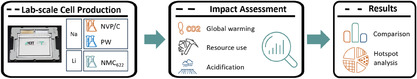

## 1 Introduction

### 1.1 Background

The shift toward renewable energies coupled with their inherent fluctuations, as well as the mobility transition, has driven a growing need for energy storage capacity.^[^ ^1^, ^2^ ^]^ Electrochemical storage plays a central role, and lithium‐ion batteries (LIBs) are currently the most prevalent option.^[^ ^2^ ^]^ However, LIBs mostly rely on critical raw materials like lithium, nickel, natural graphite, and cobalt. Although the development of lithium‐iron phosphate (LFP) batteries has partly addressed this by eliminating the need for nickel and cobalt, LFPs still require the critical materials lithium and natural graphite,^[^ ^3^ ^]^ prompting the exploration of alternative technologies.^[^ ^4^ ^]^ Battery technologies based on sodium, magnesium, aluminum, calcium, potassium, or chlorine are emerging as potential substitutes.^[^ ^5^ ^]^ Of these options, sodium‐ion batteries (SIBs) are considered to be the biggest competitor for LIBs in the near future and are already available in the market.^[^ ^6^, ^7^ ^]^

**Figure 1:**
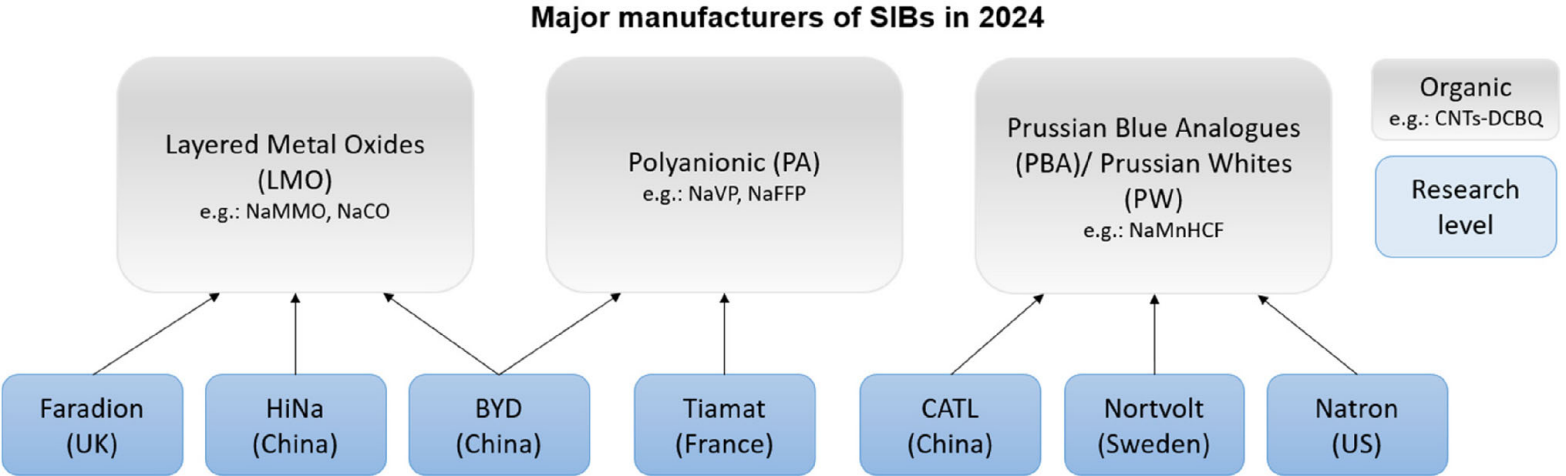
Landscape of SIB manufacturers. **NaMMO**, sodium manganese magnesium oxide; **NaCO**, sodium cobalt oxide; **NaVP**, sodium vanadium phosphate; **NaFFP**, sodium iron phosphate; **NaMnHCF**, sodium manganese hexacyanoferrate; and **CNTs‐DCBQ** (dicyanobenzoquinone on carbon nanotubes).

For SIBs, a number of types of cathode materials are under development, the three most prominent being: layered metal oxides (LMO) such as sodium manganese oxide (NaMO), polyanionic (PA) materials, such as sodium vanadium phosphate (NVP/C), and Prussian blue analogs (PBA)/ Prussian whites (PW).^[^ ^8^ ^]^ There are also organic cathode materials for SIBs, such as dicyanobenzoquinone on carbon nanotubes (CNTs‐DCBQ) but these are less widely used and are currently at a research level.^[^ ^9^ ^]^ Among these, PBAs tend to have a lower environmental impact and are more cost‐effective on a material level than other alternatives because they do not use expensive or toxic raw materials, such as cobalt or vanadium.^[^ ^6^ ^]^ However, the various manufacturers of SIBs favor different cell chemistries. The major players currently available on the market, Faradion (UK‐based), CATL (China), Tiamat (France), HiNa (China), Northvolt (Sweden), and Natron (US), produce mainly LMO, PBA, and PA cathodes, as shown in **Figure** **1**.^[^ ^10^, ^11^ ^]^ The Chinese manufacturer BYD even plans two different SIB cell types: LMO as a high power cell and PA as a high energy cell,^[^ ^12^ ^]^ and has announced a planned production capacity for SIBs of 30 GWh annually in the near future.^[^ ^13^ ^]^

Generally, SIBs offer several advantages over LIBs, the main argument for this being that they are based on abundant rather than critical raw materials, which mitigates concerns about raw material supply risk. In addition, the widespread availability of sodium and hard carbon (HC), which is the primary anode active material for SIBs, is replacing natural graphite used in LIBs, and makes SIBs a cost‐effective alternative.^[^ ^14^ ^]^ Furthermore, SIBs have a general advantage in terms of toxicity potentials compared to LIBs.^[^ ^15^ ^]^ However, SIBs are not without drawbacks, most notably their lower gravimetric energy density. This leads to several disadvantages: larger batteries are needed to reach the same storage capacity, resulting in similar environmental impacts and costs per kWh of storage capacity to those of LIBs, as more electrolyte, packaging, and so on, is needed.^[^ ^14^, ^16^ ^]^

However, to ensure low environmental impact of SIBs in the future, assessments at regular intervals are essential, as the battery industry is a highly transformative industry, where both products and processes are constantly evolving. The comparative assessment of environmental impacts and hotspot analyses are particularly important here in order to set the right priorities for further development in terms of cell chemistry selection and environmental impact reduction. However, the number of studies assessing the environmental impact of SIBs remains limited, with only 18 identified to date. In particular, few of these studies are based on primary data, with most relying on theoretical modeling and life cycle inventory (LCI) data from a small number of sources. Furthermore, studies that do use primary data often fail to provide detailed LCIs, making them difficult for other researchers to replicate. There is also a significant lack of research based on real production processes, as most studies rely on literature values for production effort rather than direct observations or measurements.

To address these gaps, the present work provides a comprehensive comparative life cycle assessment (LCA) of two different types of SIB cells based on primary laboratory‐scale data: a PW (cathode active materials (CAM): sodium manganese hexacyanoferrate [NaMNHCF]) and a PA cell (CAM: NVP/C). In addition, the two investigated cells are compared with the mature nickel‐manganese‐cobalt (NMC) LIB cell chemistry, as they are produced in the same laboratory. The aim of this study is to provide insight into the hotspots and comparison of the environmental impacts of the evaluated SIB cells and to provide an up‐to‐date assessment of the current synthesis processes for SIB CAMs.

### 1.2 Literature Review for LCA of SIB

As part of this study, a detailed literature review was carried out using Google Scholar and Science Direct, in which all available studies on the LCA of SIBs are listed. For this purpose, the search terms ‘LCA,’ ‘life cycle assessment,’ ‘environmental impact,’ ‘environmental footprint,’ and ‘sustainability assessment’ were used in combination with the terms ‘SIB,’ ‘sodium‐ion battery,’ ‘Prussian blue analogs,’ ‘PA compounds,’ and ‘LMO’, and all studies that quantify the environmental impact of one or more SIBs were collected.

**Table** **1** summarizes the results, and to the best of the authors’ knowledge, lists all studies published to the end of 2024 that address the environmental impact of SIBs. For each study, the table indicates the year of publication, the system boundaries, the cathode and anode materials, and the electrolyte of the battery (or batteries) analyzed, as well as the data source of the underlying LCI data for the cathode. Comments or the focus of the study are provided.

**Table 1:** NaNFM: sodium nickel iron manganese oxide; HC: hard carbon; NaNMMT: sodium nickel manganese magnesium titanium oxide; NaFP: sodium iron phosphate; NaVPF: sodium vanadium phosphate; NaPBA: sodium Prussian blue analogs; LFP: lithium‐iron phosphate; NMC: (lithium) nickel manganese cobalt; VRBF: vanadium redox flow battery; NaMO: sodium manganese oxide; NaMMO: sodium manganese magnesium oxide; NaMVP: sodium manganese vanadium phosphate; NaNMC: sodium nickel manganese cobalt; NaS: sodium sulphur; VRFB: vanadium redox flow battery; and NaNMMO: sodium nickel magnesium manganese oxide.

Study	Year	System boundaries	Cathode	Anode	Electrolyte	LCI of Na‐cathode	Focus/comment
Degen et al.^[^ ^45^ ^]^	2024	Cradle to gate	NaNFM_422_	HC	NaPF_6_‐based	Theoretical model	EV Context
Yokoi et al.^[^ ^46^ ^]^	2024	Cradle to gate	NaNMMT, Na_2_Fe_2_(SO_4_)_3_	HC, Na_3_LiTi_5_O_12_	NaPF_6_‐based	^[^ 16, 17, 47 ^]^	Comparison to K‐based cells
Batuecas et al.^[^ ^21^ ^]^	2024	Cradle to gate	NaFP	Sodium	NaPF_6_‐based	Primary data	Lab‐scale, liquid, and solid SIB, coin cells
Zhang et al.^[^ ^48^ ^]^	2024	Production, use phase	NaNMMT, NaVPF, NaPBA, LFP, NMC_811_	HC (Pitch, H_2_SO_4_, Benzaldehyde)	NaPF_6_‐based	^[^ 16, 17 ^]^, NaVPF^[^ ^49^ ^]^	Prospective LCA using activity browser
Bai et al.^[^ ^50^ ^]^	2023	Cradle to gate	NaNMMT	HC	NaPF_6_‐based	^[^ ^17^ ^]^	Levelized carbon emission of storage, comparison to VRFB, LFP
Carvalho et al.^[^ ^7^ ^]^	2023	Cradle to gate	NaMO (Na_0.66_MnO_2_)	Tin (Sn) powder and carbon nanofibers (SnNC anode), only metallic Sn	NaPF_6_‐based	Primary data	Commodity LCC included
Guo et al.^[^ ^19^ ^]^	2023	Cradle to grave	Presumably NaNMMO Na_x_Ni_x_Mg_x_Mn_x_O_2_	HC	NaPF_6_‐based	Primary data (manufacturer CATL)	Comparison with LFP, EV context
Lai et al.^[^ ^51^ ^]^	2023	Cradle to gate	NaMMO, NaMVP, NaNMC, NaPBA, NaNMMT, NaS	HC	NaPF_6_‐based	^[^ 16, 52 ^]^	–
Wickerts et al.^[^ ^20^ ^]^	2023	Cradle to gate	PW (Na_2_Fe_2_(CN)_6_)	HC (1: from phenolic resin, 2: made from lignin)	1: NaPF_6_‐based 2: NaBOB	Primary data (manufacturer Altris/ Northvolt)	Resource scarcity, upscaling, data from manufacturer (LIB Gigafactory)
Merino et al.^[^ ^53^ ^]^	2023	Cradle to gate	NaNMMT, NaMMO, NaNMC, NaPBA, NaMVP	HC	NaPF_6_‐based	^[^ 16, 17 ^]^	Only reproduction of work done by Peters et al.^[^ ^16^ ^]^
Nibelius^[^ ^54^ ^]^	2023	Cradle to gate	Not disclosed	Not disclosed	Not disclosed	Primary data (manufacturer Altris/ Northvolt)	–
Baumann et al.^[^ ^6^ ^]^	2022	Screening	Not applicable	Not applicable	Not applicable	Not applicable	Only CAMs under investigation
Carvalho et al.^[^ ^22^ ^]^	2022	Cradle to gate	NaMO (Na_0.44_MnO_2_)	MXene (TiAlTiC_1.85_)	Not disclosed	Theoretical model	Lab‐scale production (coin cell)
Landi et al.^[^ ^52^ ^]^	2022	Production, Use Phase	NaS	Sodium	α, β Alumina	Not disclosed	Comparison to LFP
Peters et al.^[^ ^16^ ^]^	2021	Cradle to gate	NaNMMT, NaMMO, NaNMC, NaPBA, NaMVP	HC	NaPF_6_‐based	^[^ ^17^ ^]^	Focus on cathode
Wellings^[^ ^55^ ^]^	2021	Cradle to gate	NaNMMT	Graphite	NaPF_6_‐based	^[^ 56, 57, 58 ^]^	–
Schneider et al.^[^ ^18^ ^]^	2019	Cradle to gate	Sodium nickel cobalt manganese oxide (NaNi_1/3_Co_1/3_Mn_1/3_O_2_)	HC	NaPF_6_‐based	^[^ 17, 59 ^]^	A range of different SIB cells
Peters et al.^[^ ^17^ ^]^	2016	Cradle to gate	NaNMMT	HC	NaPF_6_‐based	Technical data sheets	Several HC precursors were assessed: sugar, starch, cellulose, organic waste, and petroleum coke

In general, it can be seen that the total number of 18 studies is relatively small, considering that the first study was published in 2016. Furthermore, with the first prototypes in applications, such as cars, and announced production capacities of more than 30 GWh for SIBs for 2025,^[^ ^13^ ^]^ the technology is at battery technology readiness level 7 (‘7. Full cell prototype, demonstration for production and application’) or even 8 (‘8. Completed cell, production qualification, and demonstration under operational environment’) as defined by Baumann et al.^[^ ^6^ ^]^ while the first steps toward commercialization of the cell technology have already been taken. Despite the small number of studies overall, the publication dates show that SIBs have attracted increasing attention at the beginning of the 2020s, as their potential to become a more environmentally sustainable, safer, and cost‐effective alternative to LIBs has been recognized.^[^ ^7^, ^17^, ^18^ ^]^

Of the 18 studies, only five are based on primary data, while the remaining 13 are based on theoretical models or literature data. It is noteworthy that the two studies by Peters et al.^[^ ^16^, ^17^ ^]^ often serve as a data source. Furthermore, not all studies based on primary data provide the LCI data on which the study is based. Only the three studies by Carvalho et al.^[^ ^7^ ^]^ Guo et al.^[^ ^19^ ^]^ and Wickerts et al.^[^ ^20^ ^]^ are based on primary data and also provide the LCIs.

**Figure 2:**
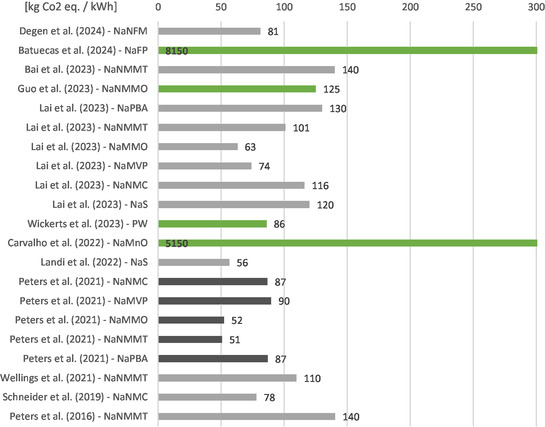
Results of LCAs on SIBs in the literature. Results are given in the impact category GWP per kWh energy capacity [kg CO_2_ eq. kWh^−1^]. Studies highlighted green: based on primary data. Study highlighted black: most‐cited study Peters et al.^[^ ^16^ ^]^

**Figure** **2** provides an overview of the results of the studies for all cell chemistries. All studies that provide the results for the impact category global warming potential (GWP) per kWh of energy capacity are listed. All other studies are either unsuitable for comparison due to a different functional unit (FU) or different system boundaries. The studies based on primary data are labeled green in the figure. The studies by Batuecas et al.^[^ ^21^ ^]^ and Carvalho et al.^[^ ^22^ ^]^ stand out with particularly high values due to the lab‐scale of production that was studied. All other studies assume industrial production using data from LIB production facilities, including Guo et al.^[^ ^19^ ^]^ and Wickerts et al.^[^ ^20^ ^]^ This means that no cell production for SIBs has been analyzed here, but data from existing LIB production facilities, mostly in GWh size, was used. In addition, the results of Peters et al.^[^ ^16^ ^]^ are highlighted as they have been reproduced many times, and thus represent a kind of benchmark. It can be seen here that all studies, with the exception of the two lab‐scale studies, give values between 50 and 140 kg CO_2_ eq. kWh^−1^, regardless of the cell chemistry analyzed. This shows that the differences in environmental impact at industrial‐scale are significantly smaller than when comparing different production scales.

In summary, there is a further need for LCA studies on SIBs, especially those that are based on primary data and make this available. Furthermore, the EU Battery Directive^[^ ^23^ ^]^ has brought a regulation into force that makes it necessary to assess the environmental impact of batteries. Even if SIBs are not yet covered by this as an emerging technology, sooner or later they will be addressed.

## 2 Experimental Section

This chapter first describes the LCA framework of this study, followed by the characteristics of the SIBs considered and the modeling approach. Finally, the reference system NMC LIB is outlined.

### 2.1 LCA Framework

LCA is a tool for assessing the environmental impact of a product or process and comparing it to other products or processes with the same function. Considering the whole life cycle, a product can be followed from its ‘cradle’, where raw materials are extracted from natural resources, through production, use phase, end‐of‐life treatment and recycling to its ‘grave’, or final disposal.^[^ ^24^ ^]^ The methodological framework for LCA is defined in the international standards ISO 14,040^[^ ^24^ ^]^ and 14,044.^[^ ^25^ ^]^ These standards divide the LCA into four stages: 1) goal and scope definition, 2) LCI analysis, 3) life cycle impact assessment (LCIA), and 4) interpretation of the results.

In accordance with the ISO standards, this study conducts an LCA of the production of two SIBs with different cell chemistries: one based on a PW material and the other based on NVP/C. The goal was to investigate the environmental hotspots of the evaluated SIB cells and compare them with each other as well as to an NMC reference cell. The study also aimed to provide an up‐to‐date assessment of the current synthesis processes for SIB CAMs. The scope is limited to the cradle to gate system boundaries and includes the entire production process, including all preceding processes, such as material extraction and background processes, with particular emphasis on the synthesis of CAMs. The FU for the assessment is set at 1 kWh of energy capacity, as storing energy represents the main function of a battery and allows, among other technical differences, assignment to different gravimetric energy densities. Furthermore, this FU is commonly used in the literature, enabling comparisons to be made between different studies.

The LCI is based on an extensive analysis of the active material synthesis, which included visiting the Battery Technology Centre at the Karlsruhe Institute of Technology (KIT), where production takes place. Data collection comprised measuring the energy consumption of different processes and material flows, leading to a detailed mass and energy balance. The production processes of the pouch cell were taken from Erakca et al.^[^ ^26^ ^]^ and adapted where necessary. Although the cited study evaluates an LIB pouch cell, it is appropriate to use the production processes for this LCA, as they are identical for lab‐scale LIBs and SIBs, except for the electrode stack drying process, which is described in detail in 2.2.4 Cell Production. For the LCIA, the open source software OpenLCA with the ecoinvent 3.9.1 database in combination with the Environmental Footprint (EF) 3.1 method was used. The recommended method for LCA by the European Commission^[^ ^27^ ^]^ is widely applied in LCA studies of batteries and includes 16 impact categories, the results of which are presented in the categories of GWP100, resource use, minerals and metals, and acidification potential in this study. While GWP is the most addressed impact category and compulsory in the frame of the Battery Regulation and the Product EF of the EU,^[^ ^23^, ^27^ ^]^ resource use is of particular interest for SIBs, being based on more abundant materials. Acidification was included as a third category to show a different angle and exhibit other main contributors. However, as all impact categories can be of interest, the detailed LCI is provided in the Supporting Information (SI), enabling LCA practitioners to reproduce the results and take on a different perspective.

### 2.2 SIBs

As indicated above, SIBs are a promising alternative to LIBs. However, what makes SIBs particularly interesting is their similar production process to LIBs. This makes SIBs a drop‐in technology, allowing an efficient conversion of an LIB production line to the production of SIBs, as no major adjustments would be necessary.^[^ ^16^, ^28^ ^]^

In the following sections, the SIBs analyzed in this study are described in more detail. The technical properties of the modeled cells, the synthesis processes of the active materials, and the cell production are outlined.

#### 2.2.1 Considered Systems – Battery Features

In this study, two distinct CAMs for SIBs are investigated: Na_3_V_2_(PO_4_)_3_/C, a PA material, hereafter referred to as NVP/C, and Na_1.8_Mn[Fe(CN)_6_]_0.95_, sodium manganese hexacyanoferrate (NaMNHCF), a PBA/PW material, hereafter referred to as PW. Both cells are produced in a lab at KIT, giving the authors access to primary process data. The NVP/C‐based cell is investigated in reference to Stüble et al.^[^ ^29^ ^]^ where it is presented as a reference cell for further SIB research activities due to its established performance characteristics. NVP/C is considered as a thermal and moisture‐stable chemistry, which also possesses a high cycle‐stability and a high rate capability, but has a rather low gravimetric capacity, and its use of vanadium as a critical and highly toxic element remains a challenge.^[^ ^30^ ^]^ The PW‐based cell is investigated building on a previous work by Büchele et al.^[^ ^31^ ^]^ where technical details on cycling of the cell as well as a postmortem analyses are presented. Generally, PWs are recognized as one of the most promising candidates for SIB cathodes due to low criticality, cost and carbon footprint,^[^ ^6^ ^]^ and therefore, represent an alternative with high potential for further development.

The processes investigated include the synthesis of the CAMs up to the formation of the finished pouch cell. Following synthesis, each CAM is processed into a cathode slurry suitable for electrode production. Different binders, as well as carbon black and graphite, are added to the respective active material. The anode slurry, produced similarly at KIT, is composed of the commercially available plant‐based HC by Kuraray as the active material, two different binders, carboxymethyl cellulose (CMC) and styrene–butadiene rubber (SBR), and carbon black. **Table** **2** presents the compositions of the slurries used for each electrode type, detailing the formulations for the NVP/C and PW‐based cathodes as well as the HC‐based anode. Details about the production processes can be found in the following subchapters. The LCI for the components is available in the Supporting Information.

**Table 2:** Electrode properties of examined battery cells. NVP/C: sodium vanadium phosphate/carbon; PW: Prussian white; HC: hard carbon; PVDF: poly(vinylidene fluoride); PAA: poly(acrylic acid); CMC: carboxymethyl cellulose; and SBR: styrene–butadiene rubber.

Slurry properties			Cathode	Cathode	Anode
Active Material			NVP/C	PW	HC
Composition	Active material	[wt%]	90.5	88	93
	Binder	[wt%]	4.5 (PVDF)	6 (PAA)	1.87 (CMC), 3.73 (SBR)
	Carbon black	[wt%]	3	4	1.4
	Graphite	[wt%]	2	2	–
Current collector			Aluminum [15 μm]		

**Figure 3:**
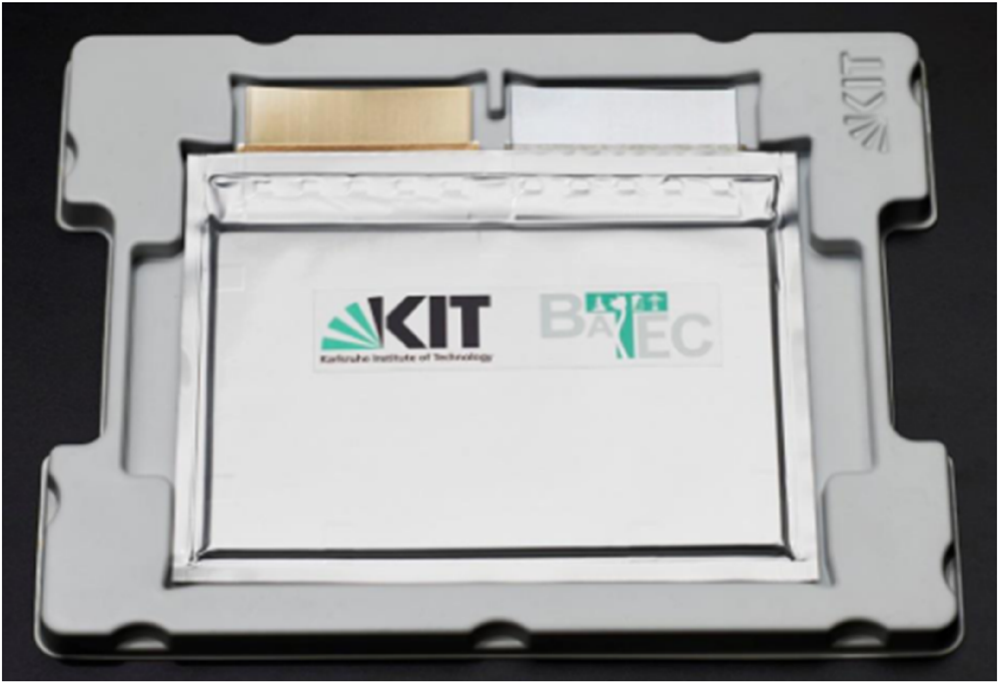
Target pouch cell format with a maximum cell stack of 6 mm. Here, a LIB is displayed.

This study focuses on three different pouch cell designs, one with PW as the CAM, alongside two cell configurations based on NVP/C, which vary primarily in the mass loading of both their cathode and anode components. This is due to the stage of development of the cells at KIT. Experiments have already been carried out with different loading thicknesses for NVP/C‐based cells, while the development of PW is less mature and only a few pouch cells are currently available. A key design criterion for all modeled cells is to maintain an electrode stack thickness of less than 6 mm. Exceeding this thickness would make assembly impractical, as the deep drawn pouch foil encasing the battery cell in this particular lab cannot accommodate a stack taller than 6 mm. **Figure** **3** shows the target cell format, displaying a LIB.

**Figure 4:**
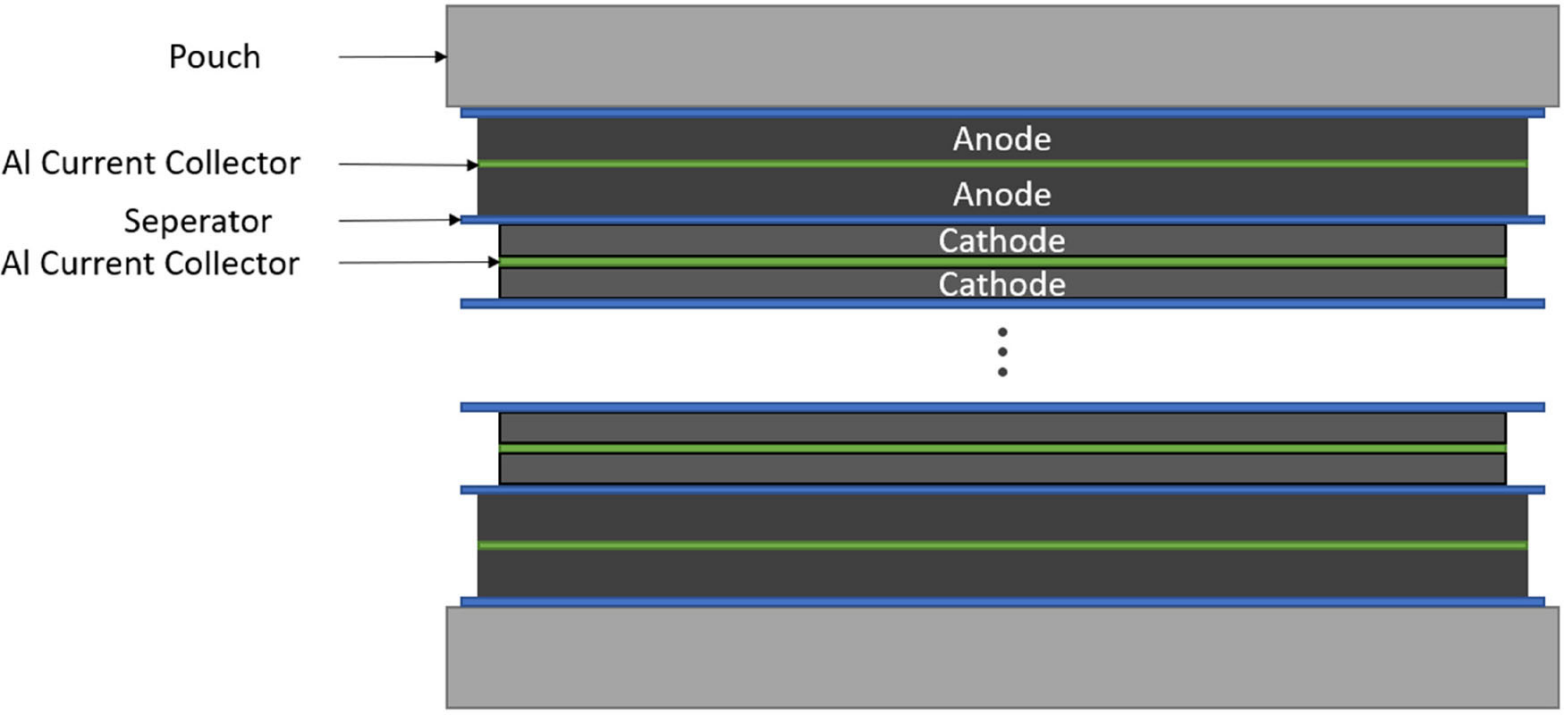
Illustration of the SIB pouch cell electrode stack.

The electrode stacks are assembled by starting with a double‐sided coated HC anode, followed by a separator sheet, a double‐sided coated cathode sheet, and another separator sheet. This four‐layer configuration is repeated until the required number of cathode sheets is included, and the stack is completed with an additional HC anode sheet. All anode and cathode sheets are double‐sided coated. Although single‐sided coatings would be sufficient on the terminal anodes, this level of customization is avoided in cell production to reduce workload and complexity. **Figure** **4** illustrates the electrode stack.

In thickness calculations, the stack height includes the double‐sided coatings on the end anode sheets, even though they lack a corresponding cathode counterpart. However, when calculating available anode capacity, these extra anode surfaces are excluded to accurately represent the usable capacity in each cell configuration. The stack thickness is calculated according to the following Equation (1).

(1) Stackthickness=cathode sheets * cathode thickness+separator sheets * separator thickness+anode sheets * anode thickness

While the electrode thickness of both the cathode and the anode depends on the mass loading of the slurry onto the current collector, the number of cathode sheets is maximized by simultaneously fulfilling the following six constraints: (1) stack thickness <6 mm; (2) anode sheets = cathode sheet + 1; (3) separator sheets = 2 × cathode sheets; (4) electrode thickness = current collector thickness + 2 × coating thickness (one side); (5) current collector thickness = 15 μm; and (6) separator thickness = 28 μm.

**Table** **3** gives an overview of the technical properties of the different pouch cells. All cells are modeled based on existing pouch cells of a similar format that have been reported in previous studies,^[^ ^29^, ^32^ ^]^ with parameters such as mass loading and the number of sheets being changed. All adjustments are kept within the feasibility constraints of KIT's laboratory.

**Table 3:** Technical properties of modeled pouch cells.

					A	B	C
Type					NVP/C	NVP/C	PW
Cathode	Mass loading			[mg cm^−2^]	22.5	10.4	16.5
	Coating thickness (one‐sided)			[μm]	185	99	146
	CAM specific capacity			[mAh g^−1^]	101.36		150
	Areal capacity			[mAh cm^−2^]	2.03	0.94	1.96
	Number of sheets				9	15	10
	Theoretical capacity before SEI formation			[Ah]	10.23	7.88	12.23
	Available capacity after SEI formation(C/10)			[Ah]	7.64	6.49	11.00
Anode	Mass loading			[mg cm^−2^]	7.85	3.94	7.85
	Coating thickness			[μm]	87	46	87
	Areal capacity			[mAh cm^−2^]	2.42	1.22	2.42
	Available capacity			[Ah]	12.85	10.75	14.28
Cell	N/P ratio				1.20	1.30	1.24
	Mass electrolyte			[g]	145.42	112.03	173.78
	Voltage (C/10)			[V]	3.11	3.09	3.2
	Energy (C/10)			[Wh]	23.78	20.04	35.21
	Stack thickness			[mm]	5.859	5.757	5.716
	Approximate gravimetric energy density			[Wh kg^−1^]	68	57	94

The three cell configurations, labeled A, B, and C, differ primarily in their cathode materials and mass loadings, with each design aiming to maximize the available energy within the 6 mm electrode stack thickness limit. All three cells are equipped with the same current collector for the anode and cathode, a 15 μm thick aluminum sheet, as well as the same separator sheets, which are composed of a ceramic layer on PET fleece. According to the primary data collection at KIT, the same electrolyte based on the conductive salt sodium hexafluorophosphate (NaPF_6_) with a composition of 1 M NaPF_6_ in ethylene carbonate (EC)/ propylene carbonate (PC) (1:1, wt%) solvent, with a density of 2.369 g ml^−1^ and a required amount of 6 mL Ah^−1^ available capacity, is used in all three configurations.

Due to the different configurations of the three cells, there is a significant difference in available energy. To obtain the energy of the cells in question, the available capacity is multiplied with the average voltage level, after the initial formation of the solid electrolyte interface (SEI). The SEI formation during the first charge–discharge cycle, builds an irreversible layer at the anode and reduces the amount of Na^+^‐ion inventory, resulting in a reduced cell capacity. It should be noted, however, that capacity and average voltage are dependent on the charge–discharge rate (C‐rate) of the cell.^[^ ^18^ ^]^ The C‐rate indicates the rate at which the battery fully discharges, expressed as an inverse fraction of an hour. Consequently, a discharge at 2C will take half an hour, at 1C one hour, and at 0.1C (also known as C/10) 10 h. It can be observed that as the rate increases, the available capacity decreases and the average voltage reached declines. In this instance, measurements of the cells with a C/10 rate were conducted for the NVP/C‐based cell, resulting in a capacity of only 82% (6.49 Ah) and 75% (7.64 Ah) of the theoretical capacity before SEI formation for the low and high mass loadings, respectively. This demonstrates that an increase in mass loading results in an increase in capacity loss due to higher C rates. In the course of this study, several discharge curves, obtained from laboratory measurement of the multilayered NVP/C‐based pouch cells, were analyzed in detail to obtain the value of average voltage available after the SEI formation. Here, the raw data of the curves was used to build an average over the charging process, leading to most accurate values for voltages. Unfortunately, the opportunity to conduct similar measurements on a PW cell was not available to the authors. However, the authors had access to primary data of C/20 discharge curves of the PW cell, allowing reasonable estimation of additional losses to be able to compare it to a C/10 discharge process.

Cell A characterizes a high cathode mass loading of 22.5 mg cm^−2^. This loading reflects the amount of slurry coated onto the aluminum current collector per centimeter squared, resulting in an area capacity of 2.03 mAh cm^−^^2^ based on the specific capacity of 101.36 mAh g^−1^ for NVP/C. The cathode coating thickness, the thickness of only the dried slurry on the current collector, amounts to 185 μm (one‐sided). The anode of Cell A has a mass loading of 7.85 mg cm^−^^2^, resulting in a coating thickness of 87 μm (one‐sided), and with a specific capacity of 332 mAh g^−1^ of the HC, provides an area capacity of 2.42 mAh cm^−^^2^. Taking into account the 15 μm thickness of both current collectors and a 28 μm thickness for each separator sheet, Cell A's stack includes nine double‐sided cathode sheets, ten double‐sided anode sheets, and 18 separator sheets to meet the 6 mm thickness constraint.

Cell B uses the same NVP/C CAM with an adjusted, lower mass loading of 10.4 mg cm^−^^2^, reducing the area capacity to 0.94 mAh cm^−^^2^. This design is based on a multilayer pouch cell model from Stüble et al.^[^ ^29^ ^]^ with the modification of double‐sided coated electrodes. The reduced mass loading results in a one‐sided cathode coating thickness of 99 μm. The anode's mass loading has been correspondingly adjusted to align with the lower cathode loading. This configuration allows for a higher number of layers, with the stack containing 15 double‐sided cathode sheets, 16 double‐sided anode sheets, and 32 separator sheets, all while maintaining the target stack thickness.

Cell C uses PW as the CAM, with a cathode mass loading of 16.5 mg cm^−^^2^. PW has a specific capacity of 150 mAh g^−1^, resulting in an area capacity of 1.96 mAh cm^−^^2^ and a one‐sided cathode coating thickness of 146 μm. The HC anode matches the configuration used in Cell A, with a mass loading of 7.85 mg cm^−^^2^ and a coating thickness of 87 μm (one‐sided), leading to an N/P ratio of 1.24 for Cell C. This configuration includes ten cathode sheets, 11 anode sheets, and 20 separator sheets.

In terms of voltage, the PW‐based cell exhibits the highest average voltage at 3.2 V, followed by Cell A and Cell B at 3.1 V. The energy output of the cells is a function of the available capacity and voltage. Once more, Cell C displays the highest values, with an energy capacity of ≈35 Wh, while Cell A and Cell B exhibit lower energy capacities of ≈24 and 20 Wh, respectively. The principal distinction between the PW‐based cell and the NVP‐based cells can be attributed to the markedly higher available capacity of 11 Ah for the 6 mm cell, resulting from the considerably higher specific capacity of the PW active material (135 mAh g^−1^ vs. 101 mAh g^−1^ for NVP/C), in conjunction with the elevated voltages attained.

#### 2.2.2 NVP/C Synthesis

**Figure 5:**
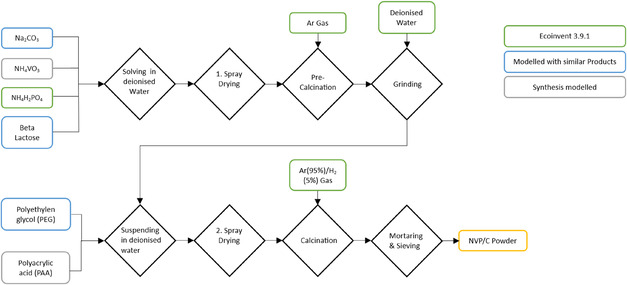
Process steps of the lab‐scale synthesis of NVP/C as CAM. The colors indicate the modeling approach for the precursor materials.

As this LCA focuses on the synthesis of the different CAMs, the processes are described in detail in this and the following section. The lab‐scale synthesis of the NVP/C CAM involves a series of carefully controlled steps, as illustrated in **Figure** **5**, which were all carried out at KIT. The following description is a summary of a complex process chain, which is described in detail in Häringer.^[^ ^33^ ^]^

The synthesis began with dissolving the precursor materials sodium carbonate (Na_2_CO_3_), ammonium dihydrogen phosphate (NH_4_H_2_PO_4_), ammonium metavanadate (NH_4_VO_3_), and β‐Lactose in deionized water. This solution was then dried in an initial spray‐drying process, followed by the precalcination under a constant argon flow. The resulting precalcined powder was subsequently ground in deionized water, at a rate of 3000 rpm, to ensure uniformity. Consequently, the suspension was further suspended in deionized water with polyacrylic acid (PAA) and polyethylene glycol as dispersing agents. The suspension undergoes a second spray‐drying process, after which the material was calcined again to achieve the desired crystalline structure. The final product was then carefully mortared and sieved to obtain a homogeneous NVP/C powder. Further details on the synthesis process, including specific conditions and parameters, can be found in Häringer or the recent study by Stüble et al.^[^ ^29^, ^33^ ^]^

#### 2.2.3 PW Synthesis

**Figure 6:**
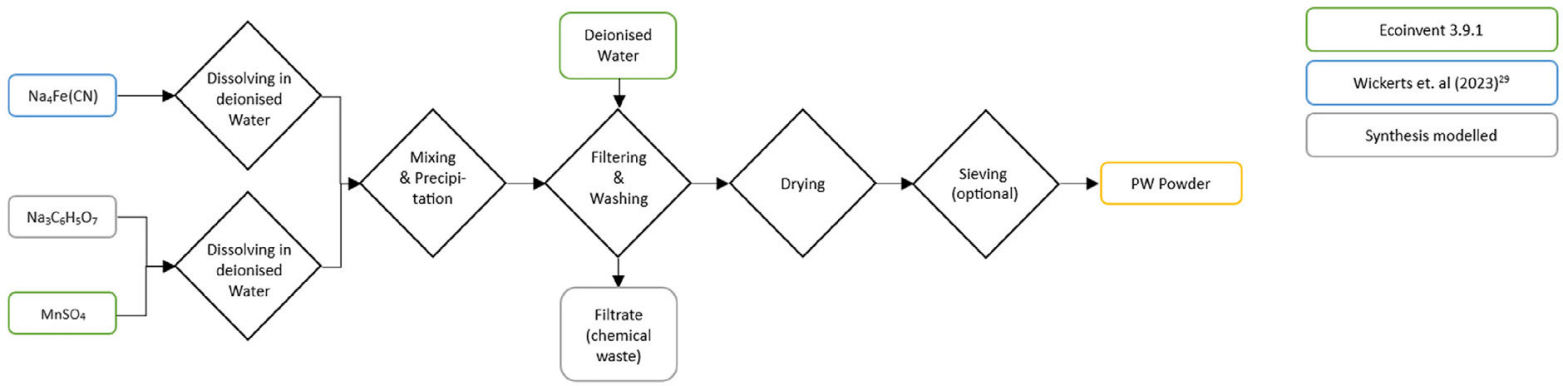
Process steps of the lab‐scale synthesis of PW as CAM. The colors indicate the modeling approach for the precursor materials.

The synthesis of PW as a CAM is relatively straightforward, involving fewer steps compared to the NVP/C synthesis. This streamlined process was also conducted at KIT and is illustrated in **Figure** **6**.

The synthesis began with dissolving the precursors in deionized water. While sodium ferrocyanide (Na_4_Fe(CN)_6_) was dissolved separately, manganese sulphate (MnSO_4_) and trisodium citrate (Na_3_C_6_H_5_O_7_) were dissolved together. The solutions were then mixed at 60 °C under a constant nitrogen flow and the precipitation reaction took place. The precipitated PW‐particles were then filtered and washed with additional deionized water to remove any impurities. The used water was then treated as chemical waste, as there is no wastewater treatment for cyanide‐containing wastewater on the lab‐scale. On an industrial‐scale, however, filtrate water treatment would be conceivable to minimize the environmental impact. Next, the filtered and washed powder undergoes a drying process for 15 h at 100 °C under a constant nitrogen flow. The produced PW powder was then optionally sieved.

#### 2.2.4 Cell Production

**Figure 7:**
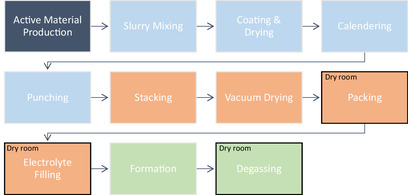
Cell production process based on Erakca et al.^[^ ^26^ ^]^ Classification: electrode production: light blue; cell assembly: orange; activating: green. Black Frame: takes place in the dry room.

The production process for all three SIB pouch cells, as well as the NMC reference cell, follows the same general steps, with a few differences. The process is modeled based on Erakca et al.^[^ ^26^ ^]^ Notably, the cells examined in that study are produced in the same laboratory at KIT, using the processes applied here, making additional measurements unnecessary. Only the CAM synthesis, slurry production, and vacuum drying process of the electrode stack differ and are therefore described in more detail. The full chain of production steps is shown in **Figure** **7**.

The process begins with the active material production for the cathode, as described in the previous subchapters, and the HC anode material, sourced from an industrial plant. These active materials were mixed with carbon black, various binders, and, in the case of the cathode slurry, graphite. The exact slurry compositions are provided in Table 2. In the next step, the slurry was coated on both sides of an aluminum current collector, which is unwound during the coating and drying process on a continuous belt dryer. Once dried, the aluminum roll was rewound into a coated electrode roll.

Next, the coated and dried electrode rolls were calendered to densify the coating, and then, punched into electrode sheets. These four steps are summarized in the following as electrode production. The double‐sided coated sheets were then the required size to be assembled into a cell. Assembly began with manually stacking the sheets, alternating coated anode, separator, coated cathode, and another separator, until the maximum stack size was reached (see Figure 4). The vacuum drying process that follows differs compared to LIB production. While LIBs can be dried at a vacuum of a few mbar and still achieve the desired properties, the electrode stacks of SIBs have to be dried at a high vacuum. As SIBs are more sensitive to water residues, a vacuum of ≤10^−3^ mbar is used here, which results in increased energy consumption. The dried stacks were transferred to a dry room to avoid moisture contamination and were packed into pouch foil casings. These casings were then filled with the electrolyte 1 M NaPF_6_ in EC/PC, also a highly moisture‐sensitive substance requiring an extremely dry assembly atmosphere.^[^ ^34^ ^]^

The final step is cell activation, which involves the formation of the SEI during the first charge–discharge cycle.^[^ ^34^ ^]^ This step results in a loss of capacity compared to the theoretical maximum, as some of the Li^+^/Na^+^ ions are bound to the electrode. Gas generation during the first charging phase creates a gas pocket, necessitating degassing as one of the final production steps.

### 2.3 LCI Modeling

#### 2.3.1 NVP/C Active Material Production

The model for the NVP/C synthesis is based on the production process conducted in the frame of Häringer,^[^ ^33^ ^]^ where a total output of 2.6 kg of active material was achieved for one batch. However, not all precursor materials used for the synthesis are available in the used database ecoinvent 3.9.1. Materials which can be found in the database are ammonium dihydrogen phosphate (NH_4_H_2_PO_4_), sodium carbonate (Na_2_CO_3_) modeled using the soda ash flow as in Peters et al.^[^ ^16^ ^]^ deionized water, argon gas, and nitrogen gas.

Materials not available in ecoinvent are modeled separately by the synthesis or represented by similar products. Ammonium metavanadate (NH_4_VO_3_) is modeled through the synthesis with ammonium chloride and sodium metavanadate, which in turn is derived from sodium hydroxide and vanadium pentoxide, flows which are available in ecoinvent. β‐Lactose is approximated using glucose for simplification due to its similar properties and negligible impact on the overall process. PAA is modeled using a polycation process of acrylic acid, and polyethylene glycol is modeled using triethylene glycol as the closest available analog with similar properties. All processes used for modeling are available in the Supporting Information.

#### 2.3.2 PW Active Material Production

The modeling of the PW synthesis is based on primary laboratory data collected during the synthesis process of 300 g conducted by one of the authors (S.B.). Among the precursor materials, manganese sulphate (MnSO_4_), deionized water, and nitrogen gas are directly available in the ecoinvent database and are used for the model. The two materials not included in ecoinvent are modeled separately based on literature and synthesis protocols. Sodium ferrocyanide (Na_4_Fe(CN)_6_) was modeled using the recent study by Wickerts et al.^[^ ^20^ ^]^ where the process is described in detail. Trisodium citrate (Na_3_C_6_H_5_O_7_) was modeled through a reaction process of citric acid and sodium hydroxide, reflecting its chemical synthesis. Furthermore, we were unable to obtain process data on the treatment of wastewater as chemical waste, as this is not done within the KIT, but carried out by an external service provider. In order to avoid any underestimation, the process with the highest environmental impact has been assumed. This is an incineration of wastewater and a subsequent thermal breakdown of the gases at 1000 ° C to avoid highly toxic gases to be released in the atmosphere. It should be mentioned, that this is a worst‐case assumption, as wastewater treatment processes with significantly lower energy requirements are available and used on higher scales. On industrial scale, the study by Wickerts et al.^[^ ^20^ ^]^ presented a precipitation reaction and reuse of the byproducts in the wastewater.

#### 2.3.3 Energy Demand and Electricity Mix

The energy demand for the production process of the battery cells is mostly derived from Erakca et al.^[^ ^26^ ^]^ as the processes for SIBs are identical to those for LIBs, with the exception of the drying process of the cell stack. In this case, the demand was specifically measured in the laboratory. The cell stacks are dried at 130 °C and ≤10^−3^ mbar, for 12 h in a vacuum dryer, requiring around 7.4 kWh for the heating and around 8 kWh for the vacuum pump. This results in a total energy demand of 15.4 kWh for the process. Given that the dryer has a maximum capacity of 20 stacks, an energy demand of 0.77 kWh per cell stack is calculated.

For the synthesis processes, most energy demands are directly measured, especially for the energy‐intensive process steps. However, when measurements are not available, the demands are calculated based on duration of the process and the power ratings of the corresponding machinery. The calcination processes show the highest energy demand with ≈70 kWh per kilogram of NVP/C produced per calcination process, followed by the spray‐drying process at 50 kWh per kilogram and process, both of which were identified as critical energy hotspots. Grinding also has a high energy demand of ≈11 kWh per kilogram NVP/C produced. All other process steps of the synthesis of NVP/C, namely mixing, solution suspension, mortaring, and sieving, show negligible energy demands of less than 1 kWh per kilogram. However, it should be noted that this lab‐scale synthesis is not optimized to minimize the energy demand, as the focus is on the material properties.

**Figure 8:**
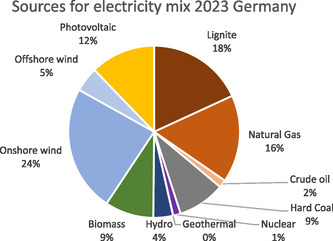
Used electricity mix in Germany in 2023 based on statistics from the German Association of Energy and Water Industries (BDEW).^[^ ^35^ ^]^

When considering the central role of energy demand in LCAs of production processes, it is crucial to use an up‐to‐date electricity mix that accurately reflects the process conditions. However, the electricity mix provided by the ecoinvent database is out‐of‐date and does not fully account for changes in recent years, such as the rise in renewable energy use, especially the increase of more than 14 GW photovoltaic electricity generation capacity in Germany in 2023. To address this, the electricity mix was modeled based on gross electricity generation in 2023 from the German Association of Energy and Water Industries (BDEW).^[^ ^35^ ^]^ The modeled mix is illustrated in **Figure** **8**, which shows that renewables account for 54% of gross electricity generation, with wind energy contributing 29% (24% onshore and 5% offshore), photovoltaic 12%, biomass 9%, hydro 4%, and geothermal less than 1%. Fossil fuels constitute 45% of the mix, including lignite 18%, natural gas 16%, hard coal 9%, and crude oil 2%. Nuclear energy represents just 1%, reflecting Germany's transition towards a nuclear‐free energy policy.

The LCI modeling of the electricity mix is based on a number of decisions to accurately map the presented data, which are briefly explained here (detail can be found in the Supporting Information). First, a grid loss factor of 5% is included to represent the transformation losses from high‐to medium‐ and medium‐to low‐voltage levels. Second, the BDEW category “other fossil energy carriers” accounts for around 3.4%, which is distributed among lignite, hard coal, and natural gas in the data presented. Third, the energy contribution from waste is equally divided between biomass and crude oil. Fourth, the distribution of onshore wind power installations is updated based on data from FA Wind^[^ ^36^ ^]^ leading to 21.2% stemming from turbines with capacities of less than 1 MW, 59.4% from turbines with 1–3 MW, and 19.4% from turbines with capacities of more than 3 MW. Similarly, the distribution of photovoltaic installations was modeled with 29% for ground‐mounted systems and 71% for rooftop installations, based on data from the Öko‐Institut.^[^ ^37^ ^]^

#### 2.3.4 Precursors and Minor Components

This subchapter describes the assumptions and specific modeling approaches used for precursor materials and minor components in the study, where exceptional. The anode precursor, HC, can have varying characteristics depending on the source of the carbon and its structural properties. HC can be derived from fossil‐based sources, as well as from biomass, leading to very different performances and environmental impacts.^[^ ^38^ ^]^ However, as this study's focus is not the synthesis of HC, the anode precursor is modeled using the processes provided in the widely cited study by Peters et al.:^[^ ^16^ ^]^ HC based on petrol coke on an industrial‐scale. Similarly, the electrolyte based on NaPF_6_ is modeled following the approach of Peters et al.^[^ ^16^ ^]^ The amount of electrolyte used is determined by the values of 4 and 6 mL Ah^−1^ of theoretical capacity for LIBs and SIBs, respectively. The separator, composed of a ceramic layer combined with a polyethylene terephthalate (PET) fleece, is modeled based on the production process outlined in a patent.

### 2.4 NMC Battery for Reference

In order to provide a reference point for the environmental impacts of the investigated SIBs, a LIB is also included in the study. Although an LFP battery would be suitable for such a comparison due to similar areas of application, an NMC‐based LIB was used. This is due to the fact that no LFP cells are manufactured in the KIT laboratory, and therefore, no primary data is available for this process. However, the NMC cell is produced on‐site in exactly the same way as the SIBs analyzed and is the subject of the underlying study by Erakca et al.^[^ ^26^ ^]^ In that study, the authors focused their analysis on the lab‐scale production processes of the cell, excluding the synthesis of the CAM, and the industrial‐scale CAM production processes described by Peters et al.^[^ ^16^ ^]^ were used. However, in order to provide a comprehensive assessment of the environmental impacts associated with lab‐scale cell production, it is essential to conduct a detailed examination of the lab‐scale synthesis process, as there are considerable differences in energy demand between the various scale levels. Moreover, Erakca et al.^[^ ^26^ ^]^ investigated a NMC_111_‐based cell, assuming equal proportions of nickel, manganese, and cobalt in the active material, and this information is now outdated. In order to account for the considerable environmental impact and elevated cost of cobalt, the composition of NMC cells currently employs a ratio of 6:2:2, 8:1:1, or 9:1:1 of nickel, manganese, and cobalt in the active material. The currently‐used ratios also lead to a significantly higher gravimetric energy density due to their high nickel content.^[^ ^39^ ^]^ In this study, the lab‐scale synthesis of an NMC_622_ active material is investigated, with the model based on the work of Mugumya et al.^[^ ^40^ ^]^ The choice of NMC_622_ can be attributed to the fact that it represents a well‐understood state of the art technology whose CAM synthesis is described in great detail in the aforementioned study. It should be noted, however, that the objective of this study is to quantify the environmental impacts of the SIBs, with the LIB serving as a reference cell. Therefore, the synthesis processes of the NMC active materials are not investigated in the same level of detail.

## 3 LCA Results

This chapter first presents the environmental impact of the synthesis of the CAMs, NVP/C composite, and PW on lab‐scale. It furthermore shows the impacts of the production of the three multilayered pouch cells: NVP‐A (high active material mass loading), NVP‐B (low active material mass loading), and PW. Then the cells are compared with the NMC reference cell and the results of three different sensitivity analyses are presented. All results are presented across three LCIA categories: GWP in kg CO_2_ eq., Resource use, minerals and metals in g Sb eq., and acidification potential in mol H^+^ eq. per kWh energy capacity (FU). The numerical results for all figures are given in the Supporting Information.

### 3.1 Lab‐Scale Production

**Figure 9:**
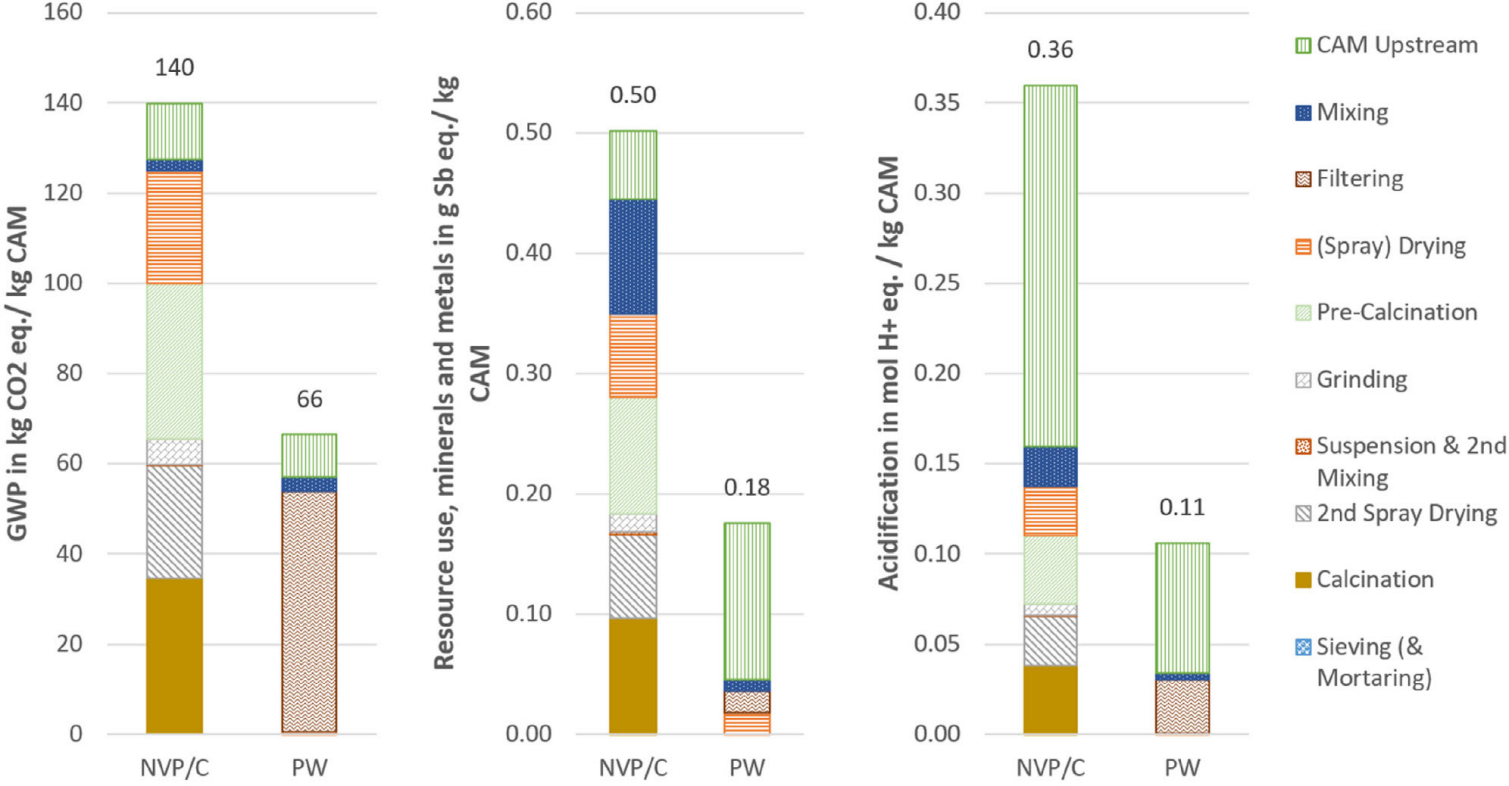
Environmental impacts of lab‐scale synthesis of the two investigated CAMs NVP/C and PW. Results are given per kg CAM produced.

**Figure** **9** illustrates the results of the lab‐scale synthesis of the two investigated CAMs, NVP/C, and PW, across three impact categories. It can be seen that the synthesis of NVP/C exhibits higher environmental impacts compared to PW, primarily due to its more complex synthesis process. The production of NVP/C involves multiple energy‐intensive steps, including two calcination and spray‐drying processes, which require high temperatures. These high energy demands lead to high environmental impacts. In contrast, PW synthesis is simpler, involving only one drying process with lower temperatures, requiring a lot less energy.

For NVP/C, the GWP impacts are evenly distributed across the production process, whereas for PW, the majority of impacts are attributed to the filtering process. This is due to the treatment of the filtrate as hazardous waste, which has a high energy demand, leading to high impacts in the category GWP. The drying process, on the contrary, causes much lower impacts for the PW synthesis, as it is a vacuum drying process at only 60 °C requiring much less energy compared to the spray‐drying processes needed for NVP/C synthesis, where a temperature of 210 °C is needed. For the resource use and acidification potential categories, the impacts are more distributed for PW synthesis, whereas for NVP/C synthesis, a similar trend as for GWP can be observed, with the notable exception that acidification potential impacts for NVP/C are predominantly driven by the use of ammonium metavanadate in the upstream material chain.

**Figure 10:**
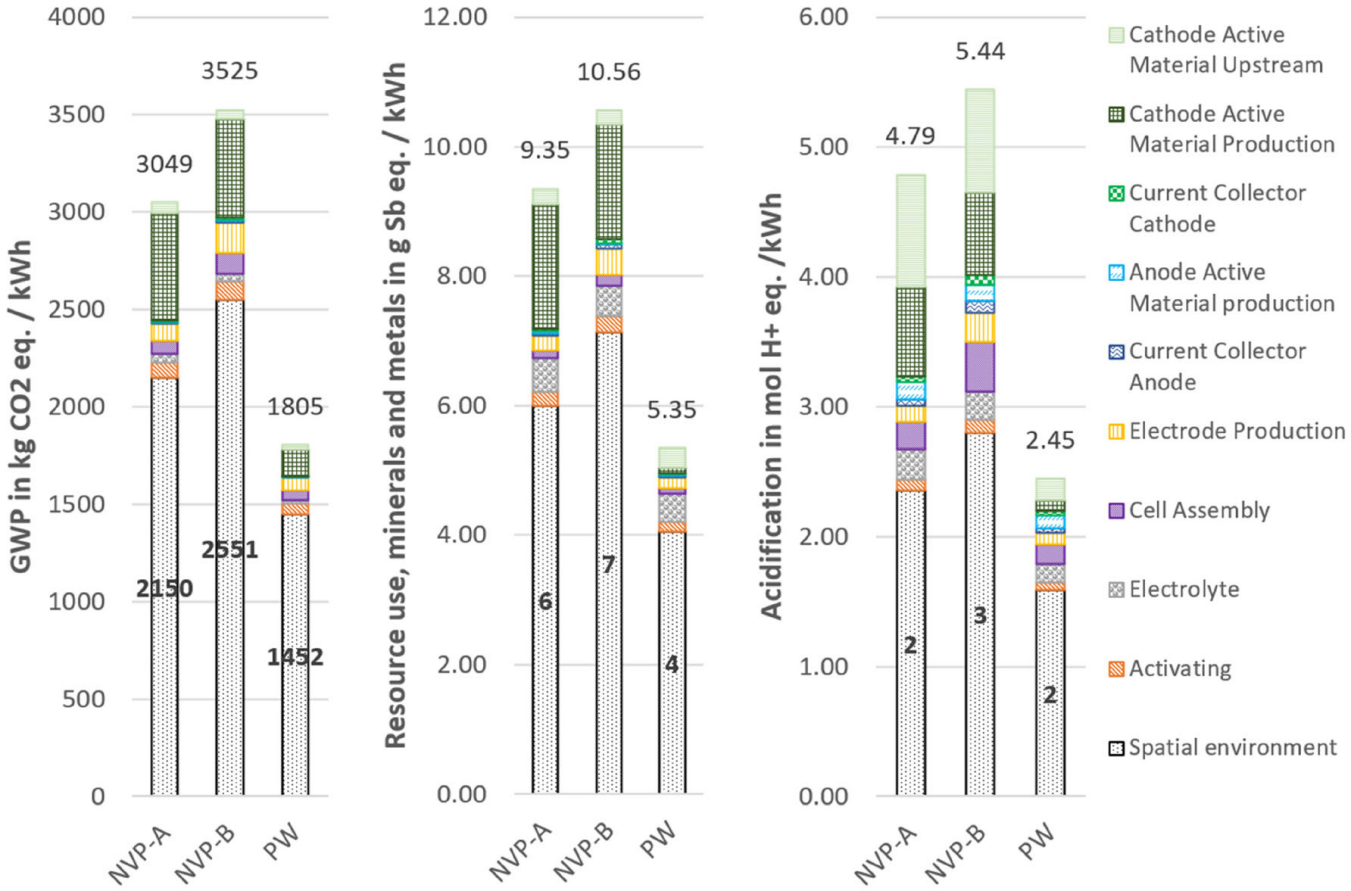
Environmental impacts of lab‐scale production of three 6 mm pouch cells. Cells A and B are based on the CAM NVP/C and the third cell on PW. Results are given per kWh storage capacity with available energies of ≈24 Wh (NVP‐A); 20 Wh (NVP‐B), and 35 Wh (PW).

**Figure** **10** shows the environmental impact of the three multilayer pouch cells studied; NVP‐A, NVP‐B, and PW. Two different loading densities were analyzed for NVP/C, whereas only one cell was analyzed for PW. This is due to the stage of development of the cells in the laboratory. Experiments with different electrode thicknesses have already been carried out for NVP/C‐based cells, while the development of PW is less advanced and only a few pouch cells are currently available. Despite the different electrode thicknesses, all cells maintain an electrode stack thickness of close to, but less than 6 mm resulting in different numbers of electrode sheets within the stacks. These differences, together with the available capacities and the C‐rate‐dependent voltage, influence the available energy capacities of the cells: the NVP‐A cell (high mass loading) delivers an available energy capacity of around 24 Wh, NVP‐B (low mass loading) 20 Wh, and the PW cell achieves 35 Wh.

In the following presentation of the results, several production steps have been grouped into categories, for better readability. The category ‘electrode production’ includes the steps of slurry production, coating and drying, calendering, and punching of each of the two electrodes, excluding the CAMs and current collectors. ‘Cell assembly’ includes stacking, vacuum drying, and packing. The category ‘activating’ includes the steps electrolyte filling, formation, and degassing. It should also be noted that for the formation, the same impacts were assumed for SIBs and the LIB, as over 90% of the energy requirement of this step can be attributed to the cycling device, equally necessary for both battery types, which in the laboratory analyzed here amounts to ≈3 kWh cell^−1^.

Since the results in Figure 10 are normalized to 1 kWh of energy as the FU, higher available energy capacities correspond to lower relative environmental impacts. This trend is evident when looking at the biggest share of impacts, those of the spatial environment. Even though they are identical in total terms for all three pouch cells, their relative impacts per kWh are lower for cells with higher available energy capacities. Therefore, the PW‐based cell has notably lower environmental impacts than the two NVP/C‐based cells across all three impact categories. Not only in relative terms but also absolute per 6 mm pouch cell, the total environmental impacts of the PW‐based cell are lower than those of both NVP/C‐based cells in all three categories. This reduction is primarily attributed to the lower environmental burden associated with cathode slurry production, stemming from the less impactful synthesis process of PW as a CAM.

It is also notable that the production of the active material for the anode has hardly any impact in the two categories GWP and resource use. This is due to the precursor of the HC, which in this case is petroleum coke. With the corresponding process by Peters et al.^[^ ^16^ ^]^ relatively very low effects are assumed. If the latest study by Liu et al.^[^ ^38^ ^]^ is followed and a bamboo‐based HC is used, the impact of the HC would be around 1500 times higher. However, it should be noted that the processes from Liu et al.^[^ ^38^ ^]^ are lab‐scale data that requires highly energy‐intensive pyrolysis, while the process from Peters et al.^[^ ^16^ ^]^ is an industrial process that requires very little energy to produce from petroleum coke.

### 3.2 Sensitivity Analysis

In addition to the cradle to gate lab‐scale analysis of the two pouch cells, three different sensitivity analyses are performed by varying the most sensitive parameters, namely the dry room throughput, the available capacity of the cells, and the electricity mix.

#### 3.2.1 Dry Room Throughput and Comparison with the Reference Cell

In order to identify the hotspots and differences of environmental impacts between the SIBs and the LIB in detail, the throughput of the dry room is increased, as it is responsible for the major share of the impacts across all impact categories and cells. The energy‐intensive dry room provides a controlled dry environment for the process steps of packing and electrolyte filling to account for the high moisture sensitivity of the components. The increase in throughput, from eight cells per 12 h to 400 cells per 12 h, is adapted from Erakca et al.^[^ ^26^ ^]^ which found that the dry room at the particular laboratory is severely oversized, and the increase in throughput mitigates the otherwise disproportionate environmental impact attributed to the dry room. More precisely, when producing only eight cells, the dry room accounts for 91.2% of the total energy demand of the pouch cell production process (excluding CAM synthesis). By increasing throughput, this share drops to ≈17%.^[^ ^26^ ^]^

**Figure 11:**
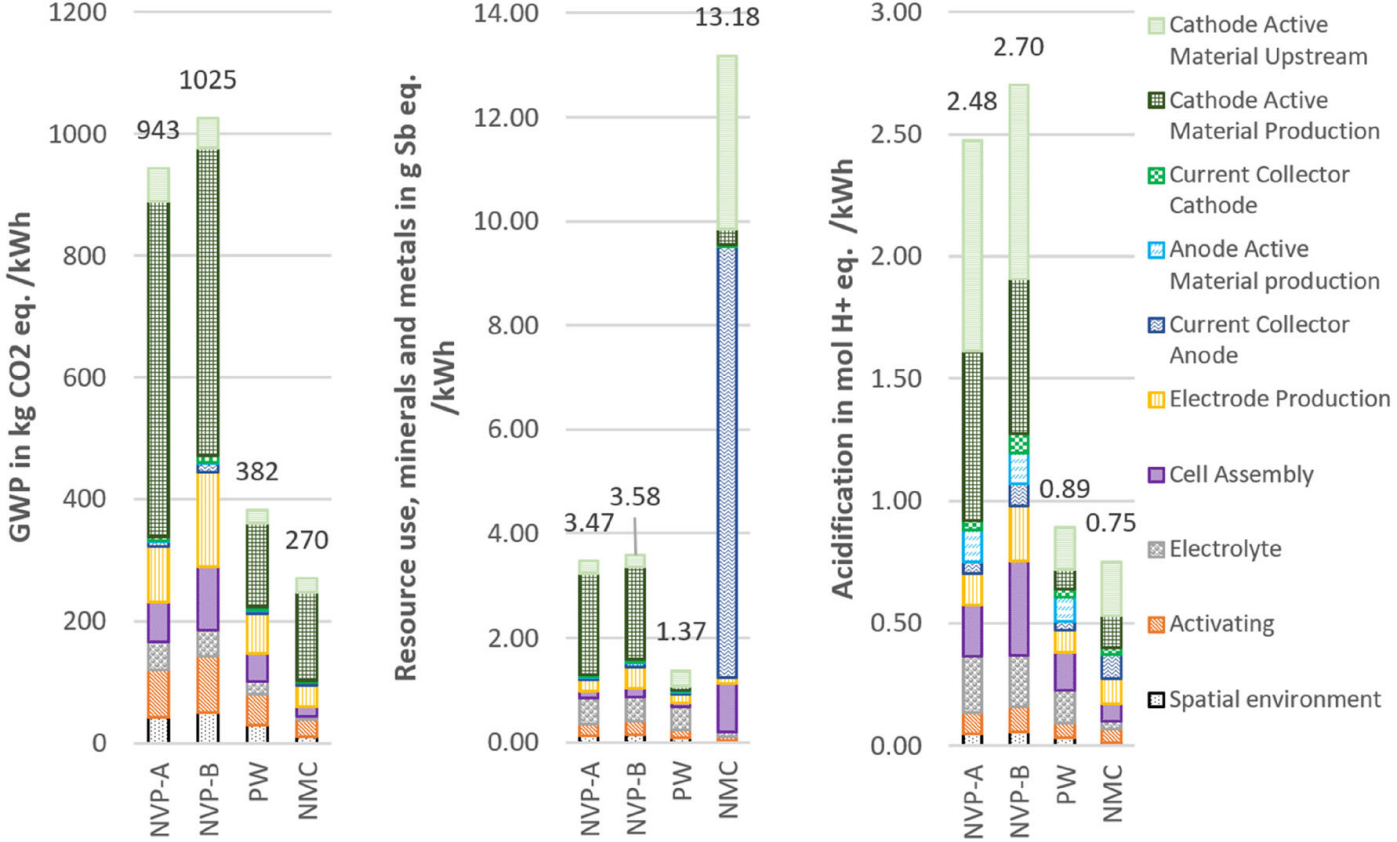
Environmental impacts of lab‐scale production of three 6 mm pouch cells, compared to NMC reference cell, with increased dry room throughput. Results are given per kWh storage capacity with available energies of ≈24 Wh (NVP‐A), 20 Wh (NVP‐B), 35 Wh (PW), and 93 Wh (NMC).

This adjustment significantly reduces the environmental impacts associated with the spatial environment per cell, and consequently, per kWh of energy (FU). Overall, the impacts decrease by 48% to 79% across all SIB cells and impact categories. **Figure** **11** presents the reduced impacts compared to those of an NMC_622_ reference cell.

For GWP, the primary contributor for all cells is the CAM production. While for PW, about 80% of the CAM production impacts are due to the filtrate treatment as hazardous waste, the impacts for NVP/C‐based and the NMC‐based cells are due to the more complex and energy‐intensive synthesis process. Among the cells, NVP‐B (low mass loading) shows the highest impacts, followed by NVP‐A, the PW cell, and finally, the NMC cell with the lowest impacts. This emphasizes the importance of the gravimetric energy density of the cells, as the FU of the assessment, and therefore, divider of the environmental impacts.

For resource use, the NMC cell exhibits the highest environmental impacts, largely due to the use of copper as the anode current collector in LIBs, compared to aluminum in SIBs, as well as the high environmental burden of cobalt and nickel. Among the SIBs, the NVP/C‐based cells again show higher impacts than PW, though the differences are smaller. For acidification potential, the same ranking as for GWP is observed. The NVP/C‐based cells have the largest impact, followed by the PW‐ and NMC‐based cell, with a similar distribution of contributing factors.

#### 3.2.2 Capacity Change due to SEI Formation

In LCAs of batteries, theoretical models are often used to assess their environmental impact. However, this carries the risk of using the theoretical capacity of the battery as the FU rather than the capacity reduced by SEI formation. In this second sensitivity analysis, the effect of using the energy capacity before and after SEI formation as FU is assessed. The FU is inversely proportional to the environmental impacts, leading to an increase in impact, when the capacity declines.

**Figure 12:**
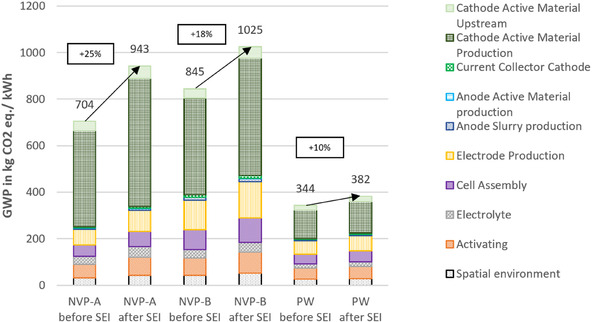
Environmental impacts of lab‐scale production of three 6 mm pouch cells with practical and theoretical capacities, including and excluding the SEI losses, respectively. Results are given per kWh storage capacity.

**Figure** **12** shows the impact when calculated with the theoretical capacities before SEI formation (left‐hand side of the two bars per cell chemistry) compared to the calculation with the available capacities after SEI formation (right‐hand side) for the impact category GWP. Higher impacts of 10%–25% for the available capacities after SEI formation show the importance of the use of measured values and the magnitude of SEI formation on environmental footprints. This is especially important for assessments of batteries in early technology readiness level, as these often use theoretical rather than measured capacity values. It can also be seen that this effect is higher for cells with higher electrode mass loading (compare NVP‐A: −25% to NVP‐B: −18%), since the SEI formation is more severe.

#### 3.2.3 Electricity Mix

For further sensitivity analysis, an electricity mix based on only renewable energy sources is assumed for all foreground processes from active material production to cell activation. Based on the electricity mix used in this study, a distribution of 58% onshore wind, 12% offshore wind, and 30% PV was assumed. The shares correspond to the distribution of the aforementioned energy sources in the electricity mix for 2023, which is described in Section 2.3.3 Energy Demand and Electricity mix.

**Figure 13:**
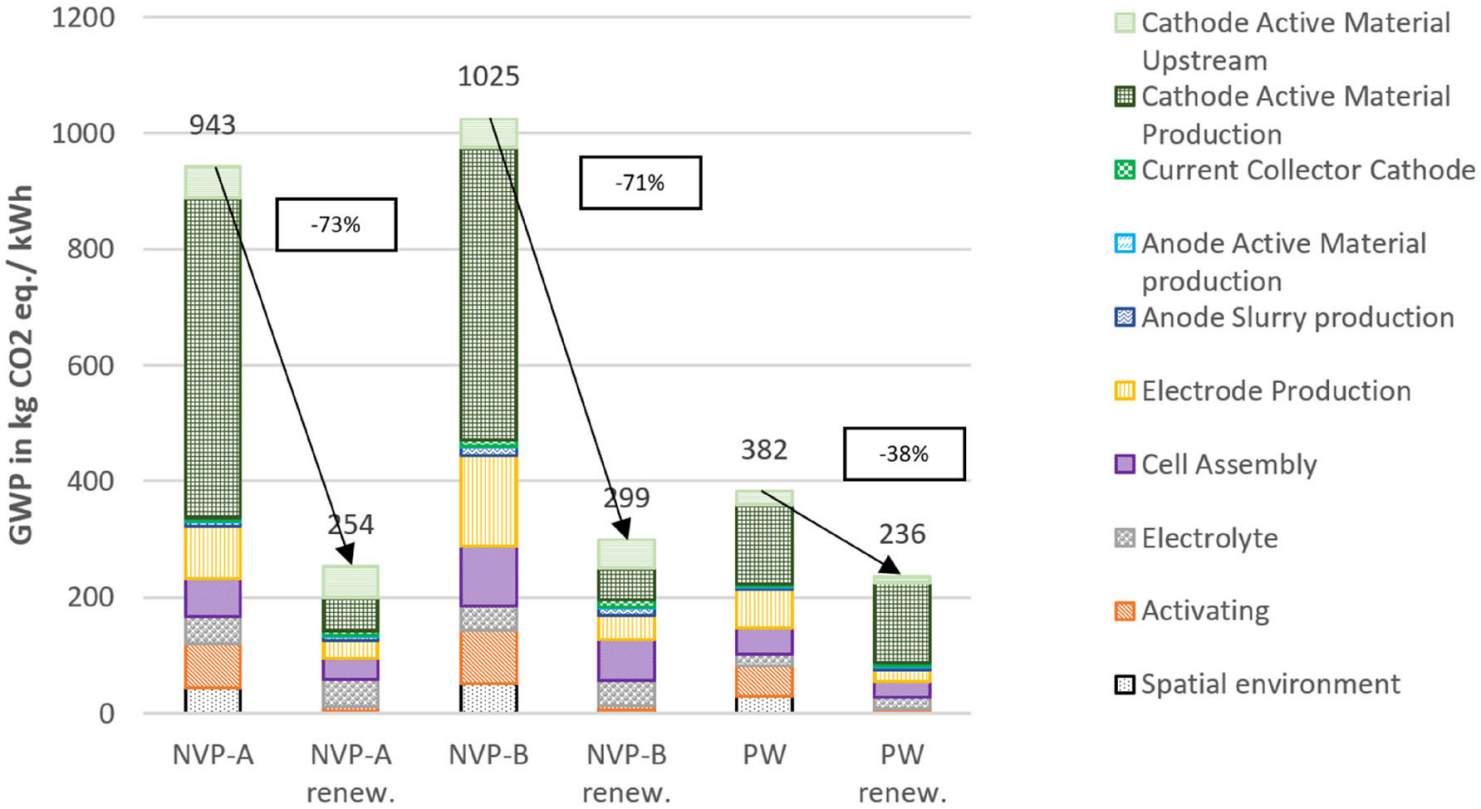
Environmental impacts of lab‐scale production of three 6 mm pouch cells using different electricity mixes: 2023 grid mix (left) and based on renewable energies (right). Results are given per kWh storage capacity.

**Figure** **13** shows the differences between the cradle to gate environmental impacts of the three investigated SIBs when using the regular electricity mix (left‐hand side of the two bars per cell chemistry) and the reduced impacts when using the electricity mix based on renewable energies (right‐hand‐side) in the impact category GWP.

It is apparent that replacing the electricity mix has a major effect on the environmental impact of producing an SIB at laboratory level. In this case, a reduction of more than 70% can be observed for the NVP/C‐based cells, while the impact of the PW cell is reduced by more than 30%. The main contributor in the category GWP for the PW cell, the treatment of the filtrate, remains unaffected by the change of electricity mix, as this process step only requires heat as energy source, but no electricity. For the NVP/C‐based cells, the reduction is particularly noticeable in the energy‐intensive production steps, namely CAM production, electrode production, activation, and the spatial environment. However, it should be noted that the environmental impact of 1 kWh from renewable energy does not lead to a reduction in the environmental impact in the category of resource use. While a reduction of over 90% can be achieved for GWP (from 0.49 to 0.04 kg CO_2_ eq.), an increase of around 90% from 1.38 to 2.62 E‐06 kg Sb eq. is recorded in the resource use category. This is primarily due to the resource consumption for the construction of PV and wind installations. The impacts in all categories for both electricity mixes used as well as the saving potentials are shown in the Supporting Information.

## 4 Discussion

The results of the conducted LCA present the hotspots of a lab‐scale SIB production and allow a comparative analysis between two NVP/C‐based SIB cells, a PW‐based SIB cell and an NMC‐based LIB cell. It is recognized that the results presented in this study represent a specific laboratory setup and synthesis route and cannot be generalized.^[^ ^38^ ^]^ Assessments at lab‐scale are not necessarily transferable to other scales and their results should not be considered definitive, but rather as a signal in sustainable technology development.^[^ ^41^ ^]^ Lab‐scale LCA is recognized as an effective tool for recommending low‐impact design of emerging technologies and materials, by identifying hotspots and reduction potentials for environmental impacts.^[^ ^42^, ^43^ ^]^ Especially in early stages of technology development, the design flexibility is high, allowing necessary changes to be made with low efforts.^[^ ^44^ ^]^ The comparison with an established technology, in this case, the NMC_622_ cell, enables both the verification of the correct order of magnitude of the results and the comparison of the hotspots for environmental impact.

The results show that the synthesis of the NVP/C CAM produces significantly higher impacts in all three categories—GWP, resource use, and acidification potential—compared to PW. The difference is mainly attributable to the more complex production process chain for NVP/C, which requires significantly higher energy inputs, in line with literature findings.^[^ ^42^ ^]^ This can be seen by looking at the contributions of the process steps with the highest share of impact; these are the steps with the highest energy demand (both calcination and both spray‐drying processes). Another factor, especially notable in the impact category of acidification potential, are the higher upstream impacts, mainly stemming from the use of vanadium. From this point of view, the substitution of vanadium or the use of other PA compounds is expedient in order to reduce the environmental impact. In contrast, in the case of PW synthesis, the treatment of the filtrate, which must be disposed of as hazardous waste on lab‐scale, plays the central role. This process produces very high direct CO_2_ emissions, which account for ≈80% of the GWP. However, the current process models the environmentally worst case as it includes the treatment of the full washing water, instead of separating the filtrate from the water, so that only the filtrate, and thus, a much smaller mass has to be disposed of as hazardous waste. One possible treatment is the precipitation process, as it is modeled in the study by Wickerts et al.^[^ ^20^ ^]^ for industrial scale. This offers huge potential for the transition from lab‐to‐industrial‐scale production in terms of environmental impact.

Regarding hotspots for the environmental impacts of cell production, first the dry room has to be considered. In this particular case, the impact attributable to the energy demand of the dry room takes up a significant share of the overall impacts. This is due to the oversized nature of the dry room and its high energy consumption. However, the impact can be significantly reduced to appear proportionate by increasing the throughput from 8 to 400 cells, as shown in the results of the first sensitivity analysis. After the dry room, the CAM synthesis accounts for the largest share of the environmental impact, followed by the electrode production. Consequently, the impacts attributable to cell activation, cell assembly, and electrolyte, in different order but of similar magnitude depending on the cell and impact category, must be mentioned. The impact of current collectors and anode active material production is relatively small. This shows that in the production of SIBs, particular attention must be paid to CAM production, especially the energy‐intensive steps, and that for the further development of SIBs, a cell chemistry should be chosen that enables simple CAM synthesis from the point of view of environmental impact.

Comparing the impacts of the SIB cells with those of the NMC LIB cell, the NVP‐B cell (low mass loading) has the highest impacts, followed by NVP‐A, the PW cell, and finally, the NMC cell, with the lowest impacts in the GWP and acidification potential categories. The environmental impacts are inversely proportional to the energy capacities of the cells. The NMC cell has a more than 2.5 times higher energy capacity than the PW cell and about four times higher than the NVP/C cells (NMC: 93 Wh, PW: 35 Wh, and NVP/C: 20–24 Wh). This is attributable to the high mean voltage and specific capacity. In terms of resource use, however, the trend differs for this particular lab‐scale production process chain. The NMC cell has impacts that are 4–5 times higher than those of the SIB cells. This is mainly due to the use of copper as the current collector for the anode in LIBs, whereas aluminum is used in SIBs, as sodium does not form unwanted alloys with aluminum at room temperature. The NMC cell's reliance on rare metals, such as nickel and cobalt, for the CAM also drives up its resource use impacts. This shows that avoiding the use of critical raw materials is particularly important in this category, whereas, it is not decisive in the GWP and acidification potential categories.

The study shows once again that a major share of the environmental impact of energy‐intensive manufacturing processes is attributable to the impact of the energy source, in this case, the electricity generation. Therefore, by using an up‐to‐date electricity mix process based on the latest statistics, this work ensures a realistic and accurate modeling of the environmental impacts. The importance of using accurate energy mixes becomes clear when looking at the results of the sensitivity analysis in this work. Here, it was shown that the use of exclusively renewable energy sources for the electricity mix can achieve an environmental impact reduction of 32%–73% in the GWP category compared to the use of the actual electricity mix.

In addition, the results of the sensitivity analysis highlight the significant differences in impact when calculations are based on theoretical values before SEI formation versus practical data after SEI formation. A difference of up to 25% could lead to incorrect conclusions and recommendations. The differences are attributable to the loss of cell capacity due to irreversible losses during the formation of the SEI in a first charge and discharge cycle, among others. Additionally, when obtaining practical data for batteries, it should be clearly stated that parameters, such as the discharge rate (C‐rate), have a significant influence on the available capacity as well as the reached voltage of a battery cell. While these factors are often overlooked in existing LCA studies, their importance cannot be overstated. For example, Schneider et al.^[^ ^18^ ^]^ showed that varying the C‐rate from C/4 to 1 °C can lead to a 90% increase in environmental impact. These findings emphasize the need to include practical data, such as discharge curves in environmental assessments, rather than relying solely on theoretical values. Particularly in comparative assessments, it is important to ensure that results are based on similar assumptions in this respect and that these assumptions are made available for future comparisons. A robust understanding of real‐world performance and production conditions is essential to capture the true EF of battery technologies and to guide sustainable innovation in the field.

### 4.1 Comparison with Existing Studies

**Figure 14:**
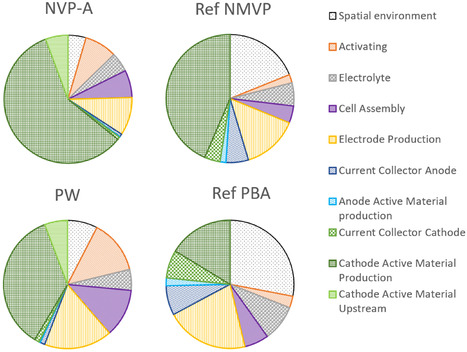
Shares of GWP impacts of cell production per kWh energy capacity. Reference study: Peters et al.^[^ ^16^ ^]^ **NVP‐A**: sodium vanadium phosphate; **PW**: sodium Prussian white; **NaMVP**: sodium manganese vanadium phosphate; **NaPBA**: sodium Prussian blue analog.

Comparing the results of this study with existing research is challenging due to significant differences in assumptions and frameworks. Most of the studies presented in the literature review focus on industrial‐scale production, which makes comparisons of absolute impacts inappropriate, but simultaneously highlights the novelty of this lab‐scale research. The two lab‐scale studies by Batuecas et al.^[^ ^21^ ^]^ and Carvalho et al.^[^ ^7^ ^]^ each assess coin cells, and consequently, reported very high impacts per kWh (8150 and 5150 kg CO_2_ eq. kWh^−1^, respectively), making a direct comparison of absolute values unfeasible. **Figure** **14** shows a comparison of the shares of the different process steps used at industrial‐scale to the shares obtained in this study.

Here, the shares of the highly cited study by Peters et al.^[^ ^16^ ^]^ for the sodium manganese vanadium phosphate (NaMVP)‐based cell are compared with the shares of impacts for the NVP‐A cell as well as the sodium PBA (NaPBA)‐based cell with the PW‐based cell from this study. When comparing both vanadium‐containing battery cells, it can be seen that for lab‐scale production, the share of CAM production dominates, exceeding that of industrial‐scale cells. This is due to the higher energy requirements per battery and per kWh at lab‐scale. A similar, more extreme observation can be made for the PW‐based cell. The treatment of the filtrate as hazardous waste plays the biggest role here, which is not optimized on a lab‐scale. An optimization in the form of a wastewater treatment process would be conceivable on an industrial‐scale. As the reference work is based on a theoretical model and does not depict any real processes, the distribution of impacts is more even. This highlights an important point when using theoretical models: it must be ensured that the process efforts are included as specifically and accurately as possible and that it is not just the material efforts that are taken into account.

A comparison can also be made at the CAM synthesis level. In the study by Rey et al.^[^ ^42^ ^]^ ten NVP‐based cathodes from the existing literature were investigated. Results for the production of half‐cells of 539.8–1622.1 kg CO2 eq. kWh_cathode_^−1^ were found. These are consistent with the results found in this study of 565–612 kg CO2 eq. kWh_cap_^−1^ for cathode production.

## 5 Conclusion

In this study, a comparative LCA of the laboratory production of two SIB cells with different cell chemistries—NVP/C (a PA material) and NaMnHCF (a Prussian blue analog/PW)—was performed to identify the hotspots for their environmental impacts. It should be mentioned that the results should be considered as indicative to support further technology design. The results show that NVP/C‐based pouch cells have higher impacts than PW‐based cells in all environmental categories. This difference is mainly due to the more energy‐intensive CAM production for NVP/C and its lower gravimetric energy density. Specifically, the lab‐scale synthesis accounts for about 50%–60% of the total impacts of the pouch cell with the increased throughput in the GWP category. The assessment was conducted within the cradle to gate system boundaries, as new primary data on production processes could be presented. For future research, an assessment of the use‐ and end‐of‐life phase would be of great interest as the cell chemistry‐specific characteristics, such as the high cycle‐stability of NVP/C, could be taken into account.

This study clearly demonstrates that streamlining and simplifying synthesis processes, while minimizing energy intensity, can be a game changer in terms of the environmental impact of battery manufacturing. It is one of few studies in the literature to show the influence of assessing the environmental impact with real battery capacities after cycling, as SEI formation can reduce capacities, and therefore, increase impacts per kWh by up to 25% compared to using theoretical values. This highlights the need for future studies to incorporate actual performance data and consistent discharge rate assumptions, which has often been neglected, while specifying the assumptions to make results more comparable in the future.

Both SIB pouch cells analyzed in this study show environmental impacts in the same order of magnitude as the benchmark NMC‐based LIB, chosen due to data availability for the specific production route and facility investigated, and therefore, produced at the same lab‐scale. While the NMC cell has lower impacts than both the NVP/C cells and the PW cell in the categories GWP and acidification potential, the PW cell has the potential to outperform the NMC cell despite the lower gravimetric energy density, when optimizing the filtrate treatment process. This issue remains unresolved and is strongly recommended to be investigated in future studies due to its high impact. In the category resource use, the NMC cell shows higher impacts than both SIBs, as it is significantly impacted by the use of copper as the anode current collector and the reliance on nickel and cobalt in the CAM. In future work, additional comparisons with other reference batteries, such as LFP‐based LIBs, which are more similar to SIBs in terms of performance, would be of interest. Additionally, by providing the full LCI, this work enables future studies to build upon the data and scale up the production processes to potentially assess the environmental impacts on industrial scale.

This study is one of the few to provide primary LCI data on the production processes for SIBs. Although this study is based on a specific lab‐scale production setup, cell design, and synthesis route, which limits its general applicability and does not represent the absolute impact of an industrial‐scale production process, lab‐scale assessments are recognized as an effective environmental consultancy tool for new technologies and materials.^[^ ^42^ ^]^ Lab‐scale results such as these can enable researchers to identify hotspots and optimize cell design and production routes, ultimately contributing to a more sustainable energy storage technology.

## Conflict of Interest

The authors declare no conflict of interest.

## Supporting information

Supplementary Material

## Data Availability

The data that support the findings of this study are openly available in Zenodo at https://doi.org/10.5281/zenodo.14811329, reference number 14811329.
