# Supplementary material for: Comparative Life Cycle Assessment of Prussian White and NVP/C‐Based Sodium‐Ion Batteries Based on Primary Laboratory Data
Source: ChemSusChem. 2025 Aug 19;18(18):e202500268. doi: 10.1002/cssc.202500268 (PMC12456382; doi:10.1002/cssc.202500268)
Supplement: Supplementary file 1 — Supplementary Material [file CSSC-18-e202500268-s001.pdf]

Supporting Information 1

Comparative Life Cycle Assessment (LCA) of Prussian White and NVP/C based Sodium-Ion Batteries based on Primary Laboratory Data

Jasper, Friedrich B.[1,2]*; Baumann, Manuel J.[1]; Ersoy, Hüseyin[1]; Smith, Anna[3]; Büchele, Sebastian[3]; Bohn, Nicole[3]; Binder, Joachim R.[3]; Neuhaus, Dirk Holger[2,4]; Weil, Marcel[1,5]

[1] Institute for Technology Assessment and Systems Analysis (ITAS), KIT, Karlsruhe, Germany

[2] Department of Sustainable Systems Engineering (INATECH), University Freiburg, Germany

[3] Institute for Applied Materials (IAM), KIT, Karlsruhe, Germany

[4] Fraunhofer Institute for Solar Energy Systems (ISE), Heidenhofstrasse 2, 79110, Freiburg, Germany

[5] Helmholtz-Institute for Electrochemical Energy Storage (HIU), KIT, Ulm, Germany

This supporting document contains additional information about the numerical results behind the figures, the sensitivity analyses and the electricity mix used.

Table of Content

1 Numerical results 2
1.1 Figure 2 2
1.2 Figure 9 3
1.3 Figure 10 4
1.4 Figure 11 5
1.5 Figure 12 6
1.6 Figure 13 6
2 Sensitivity Analysis (SEI formation): 7
3 Sensitivity Analysis (electricity mix): 9
4 Impacts of electricity mix 11

1 Numerical results

1.1 Figure 2

Cell Chemistry	kg CO2 eq. / kWh
This Study - NVP-A	943
This Study - NVP-B	1025
This Study - PW	382
Degen et al. (2024) - NaNFM	81
Batuecas et al. (2024) - NaFP	8150
Bai et al. (2023) - NaNMMT	140
Guo et al. (2023) - NaNMMO	125
Lai et al. (2023) - NaPBA	130
Lai et al. (2023) - NaNMMT	101
Lai et al. (2023) - NaMMO	63
Lai et al. (2023) - NaMVP	74
Lai et al. (2023) - NaNMC	116
Lai et al. (2023) - NaS	120
Wickerts et al. (2023) - PW	86
Carvalho et al. (2022) - NaMnO	5150
Landi et al. (2022) - NaS	56
Peters et al. (2021) - NaNMC	87
Peters et al. (2021) - NaMVP	90
Peters et al. (2021) - NaMMO	52
Peters et al. (2021) - NaNMMT	51
Peters et al. (2021) - NaPBA	87
Wellings et al. (2021) - NaNMMT	110
Schneider et al. (2019) - NaNMC	78
Peters et al. (2016) - NaNMMT	140

1.2 Figure 9

	GWP [kg CO2 eq.]		Resource use [g Sb eq.]		Acidification [mloc H+ eq.]	
	**NVP/C**	**PW**	**NVP/C**	**PW**	**NVP/C**	**PW**
Sieving (& Mortaring)	7.36E-02	6.17E-03	2.06E-04	1.72E-05	8.08E-05	6.78E-06
Calcination	3.46E+01	0.00E+00	9.65E-02	0.00E+00	3.79E-02	0.00E+00
2nd Spray Drying	2.49E+01	0.00E+00	6.96E-02	0.00E+00	2.74E-02	0.00E+00
Suspension & 2nd Mixing	3.60E-01	0.00E+00	1.97E-03	0.00E+00	7.45E-04	0.00E+00
Grinding	5.47E+00	0.00E+00	1.53E-02	0.00E+00	6.03E-03	0.00E+00
Pre-Calcination	3.46E+01	0.00E+00	9.65E-02	0.00E+00	3.79E-02	0.00E+00
(Spray) Drying	2.49E+01	5.94E-01	6.96E-02	1.79E-02	2.74E-02	6.51E-04
Filtering	0.00E+00	5.34E+01	0.00E+00	1.79E-02	0.00E+00	2.95E-02
Mixing	2.63E+00	3.19E+00	9.52E-02	9.40E-03	2.22E-02	3.76E-03
CAM Upstream	1.24E+01	9.30E+00	5.68E-02	1.30E-01	2.00E-01	7.21E-02
**Sum**	**1.40E+02**	**6.65E+01**	**5.02E-01**	**1.76E-01**	**3.60E-01**	**1.06E-01**

1.3 Figure 10

	GWP [kg CO2 eq.]			Resource use [g Sb eq.]			Acidification [mloc H+ eq.]		
	NVP-A	NVP-B	PW	NVP-A	NVP-B	PW	NVP-A	NVP-B	PW
Spatial environment	2.15E+03	2.55E+03	1.45E+03	6.01E+00	7.13E+00	4.06E+00	2.36E+00	2.80E+00	1.59E+00
Activating	7.70E+01	9.13E+01	5.20E+01	2.14E-01	2.54E-01	1.45E-01	8.64E-02	1.03E-01	5.84E-02
Electrolyte	4.66E+01	4.26E+01	2.01E+01	5.12E-01	4.68E-01	4.45E-01	2.32E-01	2.12E-01	1.38E-01
Cell Assembly	6.51E+01	1.04E+02	4.65E+01	1.19E-01	1.66E-01	8.29E-02	2.09E-01	3.83E-01	1.54E-01
Electrode Production	9.07E+01	1.56E+02	6.56E+01	2.29E-01	4.01E-01	1.70E-01	1.25E-01	2.25E-01	9.18E-02
Current Collector Anode	7.16E+00	1.36E+01	4.61E+00	3.89E-02	7.38E-02	2.50E-02	4.79E-02	9.10E-02	3.56E-02
Anode Active Material production	1.69E+00	1.61E+00	1.26E+00	2.58E-02	2.46E-02	1.92E-02	1.31E-01	1.25E-01	9.77E-02
Current Collector Cathode	6.14E+00	1.21E+01	4.61E+00	3.33E-02	6.59E-02	2.50E-02	4.11E-02	8.13E-02	3.08E-02
Cathode Active Material Production	5.52E+02	5.04E+02	1.36E+02	1.92E+00	1.76E+00	6.91E-02	6.91E-01	6.31E-01	8.09E-02
Cathode Active Material Upstream	5.37E+01	4.91E+01	2.21E+01	2.46E-01	2.25E-01	3.10E-01	8.65E-01	7.91E-01	1.72E-01
**Sum**	**3.05E+03**	**3.53E+03**	**1.80E+03**	**9.35E+00**	**1.06E+01**	**5.35E+00**	**4.79E+00**	**5.44E+00**	**2.45E+00**

1.4 Figure 11

	GWP [kg CO2 eq.]				Resource use [g Sb eq.]				Acidification [mloc H+ eq.]			
	NVP-A	NVP-B	PW	NMC	NVP-A	NVP-B	PW	NMC	NVP-A	NVP-B	PW	NMC
Spatial environment	4.30E+01	5.10E+01	2.91E+01	1.11E+01	1.24E-01	1.47E-01	8.37E-02	3.19E-02	4.73E-02	5.61E-02	3.19E-02	1.22E-02
Activating	7.70E+01	9.13E+01	5.20E+01	2.86E+01	2.14E-01	2.54E-01	1.45E-01	6.85E-02	8.64E-02	1.03E-01	5.84E-02	5.78E-02
Electrolyte	4.66E+01	4.26E+01	2.01E+01	4.05E+00	5.12E-01	4.68E-01	4.45E-01	9.37E-02	2.32E-01	2.12E-01	1.38E-01	3.05E-02
Cell Assembly	6.51E+01	1.04E+02	4.65E+01	1.60E+01	1.19E-01	1.66E-01	8.29E-02	9.36E-01	2.09E-01	3.83E-01	1.54E-01	7.16E-02
Electrode Production	9.07E+01	1.56E+02	6.56E+01	3.55E+01	2.29E-01	4.01E-01	1.66E-01	1.04E-01	1.25E-01	2.25E-01	9.18E-02	1.01E-01
Current Collector Anode	7.16E+00	1.36E+01	4.61E+00	4.02E+00	3.89E-02	7.38E-02	2.89E-02	8.28E+00	4.79E-02	9.10E-02	3.56E-02	9.87E-02
Anode Active Material production	1.69E+00	1.61E+00	1.26E+00	3.73E-02	2.58E-02	2.46E-02	1.92E-02	1.32E-03	1.31E-01	1.25E-01	9.77E-02	2.56E-04
Current Collector Cathode	6.14E+00	1.21E+01	4.61E+00	4.28E+00	3.33E-02	6.59E-02	2.50E-02	2.32E-02	4.11E-02	8.13E-02	3.08E-02	2.86E-02
Cathode Active Material Production	5.52E+02	5.04E+02	2.40E+02	1.45E+02	1.92E+00	1.76E+00	2.54E-01	3.06E-01	6.91E-01	6.31E-01	1.53E-01	1.32E-01
Cathode Active Material Upstream	5.37E+01	4.91E+01	2.15E+01	2.15E+01	2.46E-01	2.25E-01	3.09E-01	3.33E+00	8.65E-01	7.91E-01	1.67E-01	2.17E-01

Sum	9.43E+02	1.03E+03	3.82E+02	2.70E+02	3.47E+00	3.58E+00	1.37E+00	1.32E+01	2.48E+00	2.70E+00	8.90E-01	7.50E-01

1.5 Figure 12

GWP [kg CO2 eq.]	NVP-A before SEI	NVP-A after SEI	NVP-B before SEI	NVP-B after SEI	PW before SEI	PW after SEI
Spatial environment	3.21E+01	4.30E+01	4.21E+01	5.10E+01	2.61E+01	2.91E+01
Activating	5.75E+01	7.70E+01	7.53E+01	9.13E+01	4.68E+01	5.20E+01
Electrolyte	3.48E+01	4.66E+01	3.51E+01	4.26E+01	1.81E+01	2.01E+01
Cell Assembly	4.86E+01	6.51E+01	8.55E+01	1.04E+02	4.18E+01	4.65E+01
Electrode Production	6.78E+01	9.07E+01	1.28E+02	1.56E+02	5.90E+01	6.56E+01
Current Collector Anode	5.35E+00	7.16E+00	1.12E+01	1.36E+01	4.15E+00	4.61E+00
Anode Active Material production	1.27E+00	1.69E+00	1.33E+00	1.61E+00	1.13E+00	1.26E+00
Current Collector Cathode	4.59E+00	6.14E+00	1.00E+01	1.21E+01	4.15E+00	4.61E+00
Cathode Active Material Production	4.12E+02	5.52E+02	4.16E+02	5.04E+02	1.22E+02	1.36E+02
Cathode Active Material Upstream	4.01E+01	5.37E+01	4.05E+01	4.91E+01	1.99E+01	2.21E+01
**Sum**	**7.04E+02**	**9.43E+02**	**8.45E+02**	**1.03E+03**	**3.44E+02**	**3.82E+02**

1.6 Figure 13

GWP [kg CO2 eq.]	NVP-A	NVP-A renew.	NVP-B	NVP-B renew.	PW	PW renew.
Spatial environment	4.30E+01	3.88E+00	5.10E+01	4.60E+00	2.91E+01	2.62E+00
Activating	7.70E+01	7.41E+00	9.13E+01	8.79E+00	5.20E+01	5.00E+00
Electrolyte	4.66E+01	4.66E+01	4.26E+01	4.26E+01	2.01E+01	2.01E+01
Cell Assembly	6.51E+01	3.71E+01	1.04E+02	7.06E+01	4.65E+01	2.76E+01
Electrode Production	9.07E+01	3.07E+01	1.56E+02	4.15E+01	6.56E+01	1.97E+01
Current Collector Anode	7.16E+00	7.16E+00	1.36E+01	1.36E+01	4.61E+00	5.32E+00
Anode Active Material production	1.69E+00	1.69E+00	1.61E+00	1.61E+00	1.26E+00	1.26E+00
Current Collector Cathode	6.14E+00	6.14E+00	1.21E+01	1.21E+01	4.61E+00	4.61E+00
Cathode Active Material Production	5.52E+02	5.95E+01	5.04E+02	5.44E+01	1.36E+02	1.39E+02
Cathode Active Material Upstream	5.37E+01	5.37E+01	4.91E+01	4.91E+01	2.21E+01	1.13E+01
**Sum**	**9.43E+02**	**2.54E+02**	**1.03E+03**	**2.99E+02**	**3.82E+02**	**2.36E+02**

2 Sensitivity Analysis (SEI formation):

Resource use:

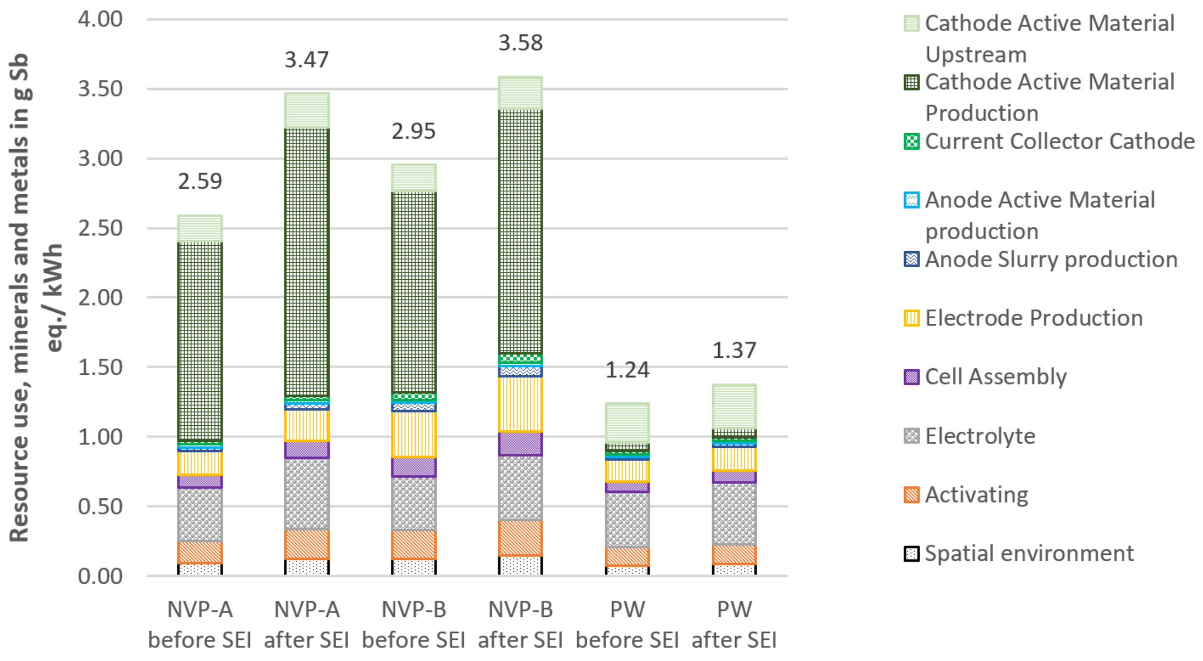

Figure i: Environmental Impacts of lab-scale production of three 6 mm pouch cells with practical and theoretical capacities, including and excluding the SEI losses, respectively. Results are given per kWh storage capacity in category Resource use.

**Resource use [g Sb eq.]**	NVP-A before SEI	NVP-A after SEI	NVP-B before SEI	NVP-B after SEI	PW before SEI	PW after SEI
Spatial environment	9.26E-02	1.24E-01	1.21E-01	1.47E-01	7.54E-02	8.37E-02
Activating	1.60E-01	2.14E-01	2.09E-01	2.54E-01	1.30E-01	1.45E-01
Electrolyte	3.82E-01	5.12E-01	3.85E-01	4.68E-01	4.01E-01	4.45E-01
Cell Assembly	8.91E-02	1.19E-01	1.37E-01	1.66E-01	7.46E-02	8.29E-02
Electrode Production	1.71E-01	2.29E-01	3.30E-01	4.01E-01	1.53E-01	1.70E-01
Anode Slurry production	2.90E-02	3.89E-02	6.08E-02	7.38E-02	2.25E-02	2.50E-02
Anode Active Material production	1.93E-02	2.58E-02	2.03E-02	2.46E-02	1.73E-02	1.92E-02
Current Collector Cathode	2.49E-02	3.33E-02	5.43E-02	6.59E-02	2.25E-02	2.50E-02
Cathode Active Material Production	1.44E+00	1.92E+00	1.45E+00	1.76E+00	6.22E-02	6.91E-02
Cathode Active Material Upstream	1.84E-01	2.46E-01	1.85E-01	2.25E-01	2.79E-01	3.10E-01
**Sum**	**2.59E+00**	**3.47E+00**	**2.95E+00**	**3.58E+00**	**1.24E+00**	**1.37E+00**

Acidification Potential:

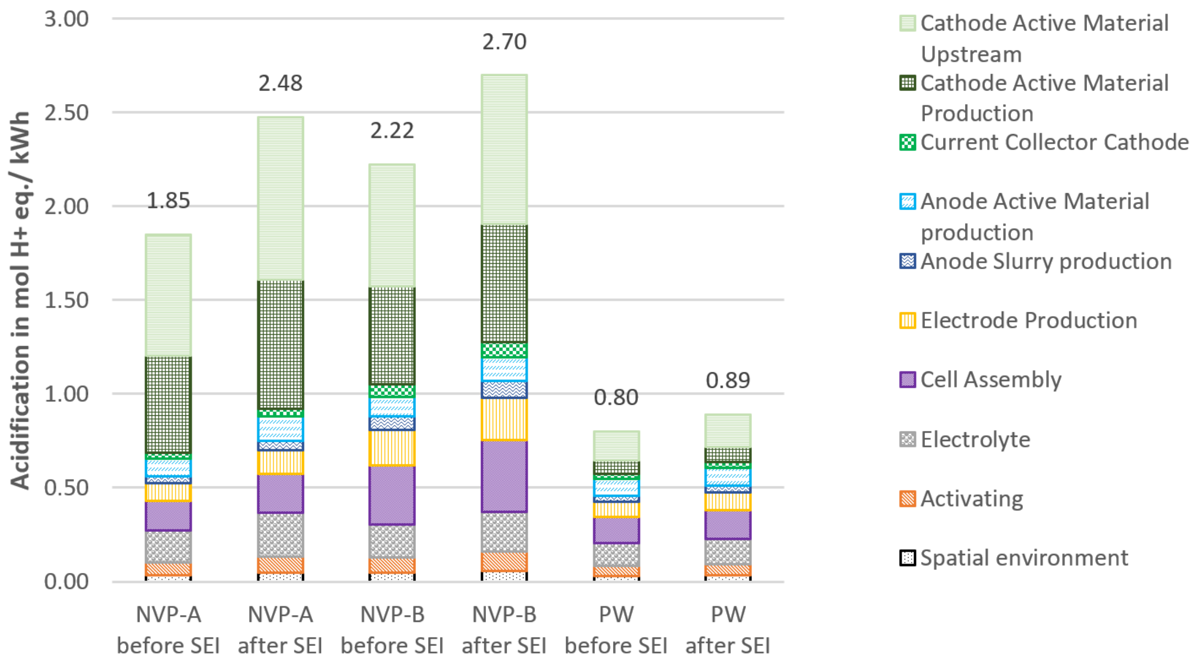

Figure ii: Environmental Impacts of lab-scale production of three 6 mm pouch cells with practical and theoretical capacities, including and excluding the SEI losses, respectively. Results are given per kWh storage capacity in category Acidification.

**Acidification [mloc H+ eq.]**	NVP-A before SEI	NVP-A after SEI	NVP-B before SEI	NVP-B after SEI	PW before SEI	PW after SEI
Spatial environment	3.53E-02	4.73E-02	4.62E-02	5.61E-02	2.88E-02	3.19E-02
Activating	6.45E-02	8.64E-02	8.45E-02	1.03E-01	5.25E-02	5.84E-02
Electrolyte	1.73E-01	2.32E-01	1.75E-01	2.12E-01	1.24E-01	1.38E-01
Cell Assembly	1.56E-01	2.09E-01	3.16E-01	3.83E-01	1.38E-01	1.54E-01
Electrode Production	9.37E-02	1.25E-01	1.86E-01	2.25E-01	8.26E-02	9.18E-02
Anode Slurry production	3.58E-02	4.79E-02	7.50E-02	9.10E-02	3.20E-02	3.56E-02
Anode Active Material production	9.82E-02	1.31E-01	1.03E-01	1.25E-01	8.79E-02	9.77E-02
Current Collector Cathode	3.07E-02	4.11E-02	6.70E-02	8.13E-02	2.78E-02	3.08E-02
Cathode Active Material Production	5.16E-01	6.91E-01	5.20E-01	6.31E-01	7.28E-02	8.09E-02
Cathode Active Material Upstream	6.46E-01	8.65E-01	6.52E-01	7.91E-01	1.54E-01	1.72E-01
**Sum**	**1.85E+00**	**2.48E+00**	**2.22E+00**	**2.70E+00**	**8.01E-01**	**8.90E-01**

3 Sensitivity Analysis (electricity mix):

Resource use:

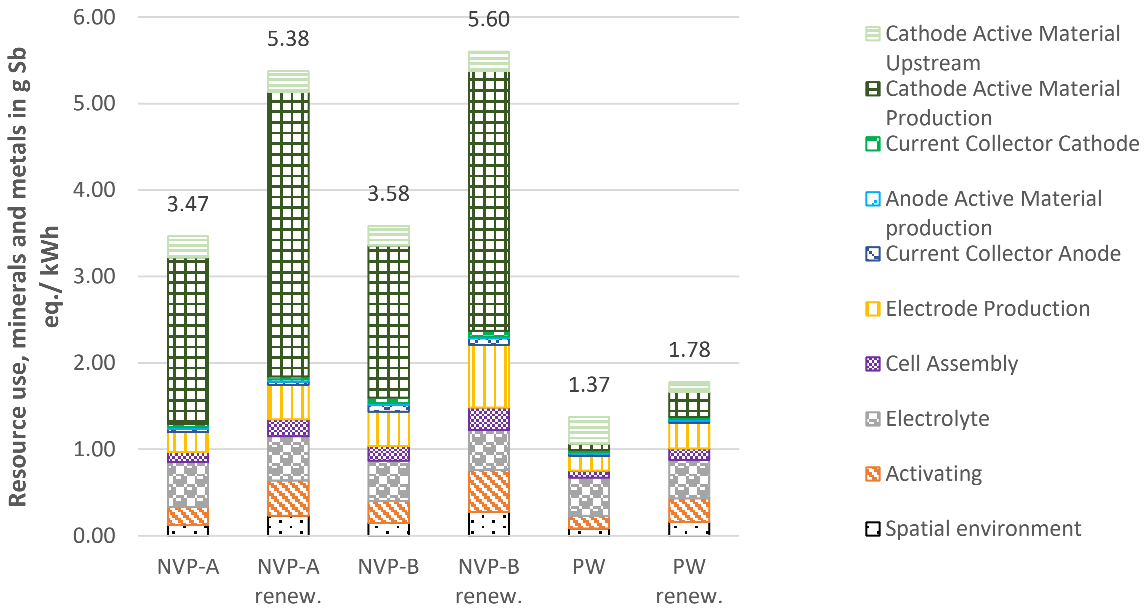

Figure iii: Environmental Impacts of lab-scale production of three 6 mm pouch cells using different electricity mixes: 2023 grid mix (left) and based on renewable energies (right). Results are given per kWh storage capacity in category Resource use.

**Resource use [g Sb eq.]**	NVP-A	NVP-A renew.	NVP-B	NVP-B renew.	PW	PW renew.
Spatial environment	1.24E-01	2.32E-01	1.47E-01	2.76E-01	8.37E-02	1.57E-01
Activating	2.14E-01	4.06E-01	2.54E-01	4.82E-01	1.45E-01	2.75E-01
Electrolyte	5.12E-01	5.12E-01	4.68E-01	4.68E-01	4.45E-01	4.45E-01
Cell Assembly	1.19E-01	1.97E-01	1.66E-01	2.58E-01	8.29E-02	1.35E-01
Electrode Production	2.29E-01	4.00E-01	4.01E-01	7.25E-01	1.70E-01	2.95E-01
Current Collector Anode	3.89E-02	3.89E-02	7.38E-02	7.38E-02	2.50E-02	2.50E-02
Anode Active Material production	2.58E-02	2.58E-02	2.46E-02	2.46E-02	1.92E-02	1.92E-02
Current Collector Cathode	3.33E-02	3.33E-02	6.59E-02	6.59E-02	2.50E-02	2.50E-02
Cathode Active Material Production	1.92E+00	3.29E+00	1.76E+00	3.00E+00	6.91E-02	2.87E-01
Cathode Active Material Upstream	2.46E-01	2.46E-01	2.25E-01	2.25E-01	3.10E-01	1.15E-01
**Sum**	**3.47E+00**	**5.38E+00**	**3.58E+00**	**5.60E+00**	**1.37E+00**	**1.78E+00**

Acidification:

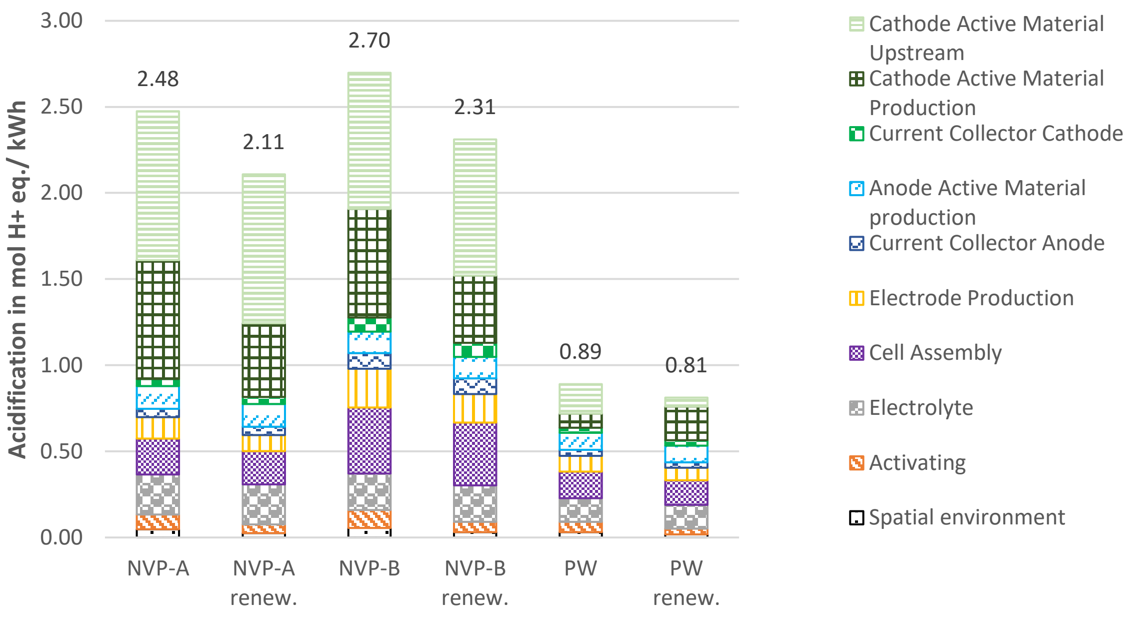

Figure iiii: Environmental Impacts of lab-scale production of three 6 mm pouch cells using different electricity mixes: 2023 grid mix (left) and based on renewable energies (right). Results are given per kWh storage capacity in category Acidification.

**Acidification [mloc H+ eq.]**	NVP-A	NVP-A renew.	NVP-B	NVP-B renew.	PW	PW renew.
Spatial environment	4.73E-02	2.64E-02	5.61E-02	3.13E-02	3.19E-02	1.78E-02
Activating	8.64E-02	4.92E-02	1.03E-01	5.84E-02	5.84E-02	3.33E-02
Electrolyte	2.32E-01	2.32E-01	2.12E-01	2.12E-01	1.38E-01	1.38E-01
Cell Assembly	2.09E-01	1.94E-01	3.83E-01	3.65E-01	1.54E-01	1.44E-01
Electrode Production	1.25E-01	9.38E-02	2.25E-01	1.65E-01	9.18E-02	7.25E-02
Current Collector Anode	4.79E-02	4.79E-02	9.10E-02	9.10E-02	3.56E-02	3.08E-02
Anode Active Material production	1.31E-01	1.31E-01	1.25E-01	1.25E-01	9.77E-02	9.77E-02
Current Collector Cathode	4.11E-02	4.11E-02	8.13E-02	8.13E-02	3.08E-02	3.08E-02
Cathode Active Material Production	6.91E-01	4.28E-01	6.31E-01	3.91E-01	8.09E-02	1.96E-01
Cathode Active Material Upstream	8.65E-01	8.65E-01	7.91E-01	7.91E-01	1.72E-01	5.25E-02
**Sum**	**2.48E+00**	**2.11E+00**	**2.70E+00**	**2.31E+00**	**8.90E-01**	**8.12E-01**

4 Impacts of electricity mix

Table 1: Environmental impacts of 1kWh electricity produced by respective mix using impact assessment method EF 3.1 (adapted).

Impact category	1 kWh Deutschland Markt low Voltage 2023 (JB) Netzverluste integriert	1 kWh Deutschland renewable (JB)	Unit	Difference market vs renew
**Acidification**	5.42E-04	3.02E-04	mol H+-Eq	-44%
**Climate change (GWP100)**	0.493997054	0.044396975	kg CO2-Eq	-91%
**Climate change - Biogenic (GWP100)**	0.007880646	1.27E-04	kg CO2-Eq	-98%
**Climate change - Fossil (GWP100)**	0.485487416	0.044189961	kg CO2-Eq	-91%
**Climate change - Land use and land use change (GWP100)**	6.29E-04	7.97E-05	kg CO2-Eq	-87%
**Ecotoxicity, freshwater**	1.45660735	0.41288826	CTUe	-72%
**Ecotoxicity, freshwater - inorganics**	1.43700232	0.39353291	CTUe	-73%
**Ecotoxicity, freshwater - organics**	0.01960503	0.019355349	CTUe	-1%
**Eutrophication, freshwater**	6.31E-04	2.60E-05	kg P-Eq	-96%
**Eutrophication, marine**	2.05E-04	5.13E-05	kg N-Eq	-75%
**Eutrophication, terrestrial**	0.001186938	5.28E-04	mol N-Eq	-55%
**Human toxicity, carcinogenic**	1.25E-10	9.96E-11	CTUh	-20%
**Human toxicity, carcinogenic - inorganics**	8.19E-11	6.41E-11	CTUh	-22%
**Human toxicity, carcinogenic - organics**	4.34E-11	3.56E-11	CTUh	-18%
**Human toxicity, non-carcinogenic**	3.98E-09	2.52E-09	CTUh	-37%
**Human toxicity, non-carcinogenic - inorganics**	3.87E-09	2.43E-09	CTUh	-37%
**Human toxicity, non-carcinogenic - organics**	1.05E-10	9.08E-11	CTUh	-14%
**Ionising radiation (human health)**	0.014447017	0.003244816	kBq U235-Eq	-78%
**Land use**	1.771632671	1.53536114	dimensionless	-13%
**Ozone depletion**	6.21E-09	2.37E-09	kg CFC-11-Eq	-62%
**Particulate matter**	5.01E-09	3.37E-09	disease incidence	-33%
**Photochemical ozone formation**	4.32E-04	1.92E-04	kg NMVOC-Eq	-55%
**Resource use, fossils**	5.722882674	0.544074325	MJ, net calorific value	-90%
**Resource use, minerals and metals**	1.38E-06	2.62E-06	kg Sb-Eq	90%
**Water use (user deprivation potential)**	0.071664042	0.049079964	m3 world eq. deprived	-32%
